# Mechanical Microenvironment in Tumor Immune Evasion: Bidirectional Regulation Between Matrix Stiffness and Immune Cells and Its Therapeutic Implications

**DOI:** 10.7150/ijbs.121356

**Published:** 2026-01-01

**Authors:** Jing Ai, Huayao Li, Minpu Zhang, Jingyang Liu, Lijuan Liu, Changgang Sun

**Affiliations:** 1 College of First Clinical Medicine, Shandong University of Traditional Chinese Medicine, Jinan 250014, China.; 2 College of Traditional Chinese Medicine, Shandong Second Medical University, Weifang, 261000, China.; 3 Faculty of Chinese Medicine, Macau University of Science and Technology, Macau 999078, China.; 4 Department of Oncology, Weifang Traditional Chinese Hospital, Weifang 261000, China.

**Keywords:** Immune evasion, Matrix stiffness, Extracellular matrix, Immune cell function, Immunotherapy.

## Abstract

Immune evasion remains a major obstacle to effective cancer immunotherapy. While the regulatory mechanisms of the tumor biochemical microenvironment are relatively well-characterized, the role of its mechanical microenvironment—particularly pathologically elevated matrix stiffness—in immune evasion remains to be fully elucidated. Immune cells, as dynamic responders within the tumor microenvironment, are not merely passive recipients of mechanical signals but also active participants in driving pathological matrix stiffening. This review focuses on the elevated matrix stiffness resulting from abnormal deposition and crosslinking of the tumor extracellular matrix, systematically elucidating how it impairs immune cell function and drives immune evasion through physical barriers and mechanotransduction. Additionally, we further propose an innovative concept: the "matrix stiffness-immune cell bidirectional regulatory axis." Dissecting this regulatory loop provides an essential mechanical perspective for understanding immune evasion and offers a conceptual framework for developing matrix-targeted strategies to enhance immunotherapy. By integrating current evidence, this review aims to clarify the role of this bidirectional axis and to identify novel therapeutic targets and strategies that may improve the efficacy of cancer immunotherapies.

## Introduction

Tumor cells evade immune surveillance and elimination by altering intrinsic properties or exploiting the host's immunosuppressive and dysregulated responses, ultimately forming clinically detectable tumors—a phenomenon termed immune evasion 1. As a major challenge in current immunotherapy, one of the reasons for the progression of immune evasion and therapeutic resistance lies in the dynamic regulation by the functional plasticity of immune cells 2. During early tumorogenesis, nascent tumor lesions can be recognized and eliminated by both adaptive and innate immune systems. As the disease progresses, however, tumor cells collaborate with the tumor microenvironment (TME) to reprogram infiltrating immune cells toward exhausted or suppressive states—characterized by an increase in dysfunctional CD8⁺ T cells, expanded regulatory T cells (Tregs) infiltration, and impaired maturation and function of dendritic cells (DCs). These changes collectively drive the transition toward an immunosuppressive TME 1, 3. Notably, the biomechanical properties of the TME, an emerging dimension of tumor biology, have been identified as pivotal drivers of immune evasion: matrix stiffness, solid stress, interstitial fluid pressure, and topological structure collectively mediate immunosuppression by impeding immune cell migration to tumor-infiltrated regions and disrupting anti-tumor immune cascades 4, 5. Among these factors, increased matrix stiffness is one of the most prominent mechanical abnormalities in malignant tumors 6. Not only does it facilitate tumor proliferation, invasion, and metastasis, but it also significantly contributes to immune evasion, thereby reducing tumor sensitivity to immunotherapeutic interventions 7-9.

Elevated matrix stiffness is a hallmark biomechanical alteration in various malignancies, including breast cancer (BC), pancreatic cancer, and hepatocellular carcinoma (HCC). Quantitative assessments of the elastic modulus reveal that tumor tissues exhibit substantially higher stiffness compared to the soft microenvironment of normal or benign tissues 10-13. For example, the average stiffness of malignant breast tumors reaches approximately 153 kPa, markedly exceeding the typical range of 40-64 kPa observed in benign lesions 10. This abnormal stiffening primarily results from excessive extracellular matrix (ECM) deposition and enhanced crosslinking 6, 14, contractile tension generated by tumor cells, and compressive stress caused by uncontrolled tumor growth 6, 15, 16. These factors act synergistically to remodel the ECM architecture, ultimately leading to increased matrix stiffness. Significantly, aberrant ECM accumulation and lysyl oxidase (LOX)-mediated crosslinking represent the principal driving forces behind matrix stiffening 17-19. This process is dynamically orchestrated by the interplay between cellular components—including tumor cells, immune cells, and cancer-associated fibroblasts (CAFs)—and acellular factors such as the hypoxic microenvironment 18, 20. Such pathologically elevated matrix stiffness also forms a physical barrier that impedes immune cell infiltration 21-23 and impairs tertiary lymphoid structure (TLS)-mediated antitumor immunity 24. This phenomenon, in which CAFs play a central role, is closely associated with the development of a desmoplastic reaction 22, 25, in which CAFs play a central role. Upon activation, CAFs undergo extensive proliferation and secrete abundant ECM components, contributing to enhanced ECM synthesis and remodeling during desmoplasia 26. Consequently, CAFs, stromal cells, and the ECM converge to form dense connective tissue that envelops tumor nests 27, 28. This physical barrier compromises immune cell trafficking to the tumor site and disrupts the formation of TLSs 24. TLSs are lymph node-like aggregates that serve as critical hubs for the initiation and maturation of antitumor immune responses and are generally associated with favorable prognosis in multiple cancers 29. Nonetheless, excessively high intratumoral matrix stiffness may disrupt TLS-driven antitumor immunity by compressing the extracellular space and restricting immune cell motility, thereby ultimately promoting immune evasion 24.

**Figure 1:**
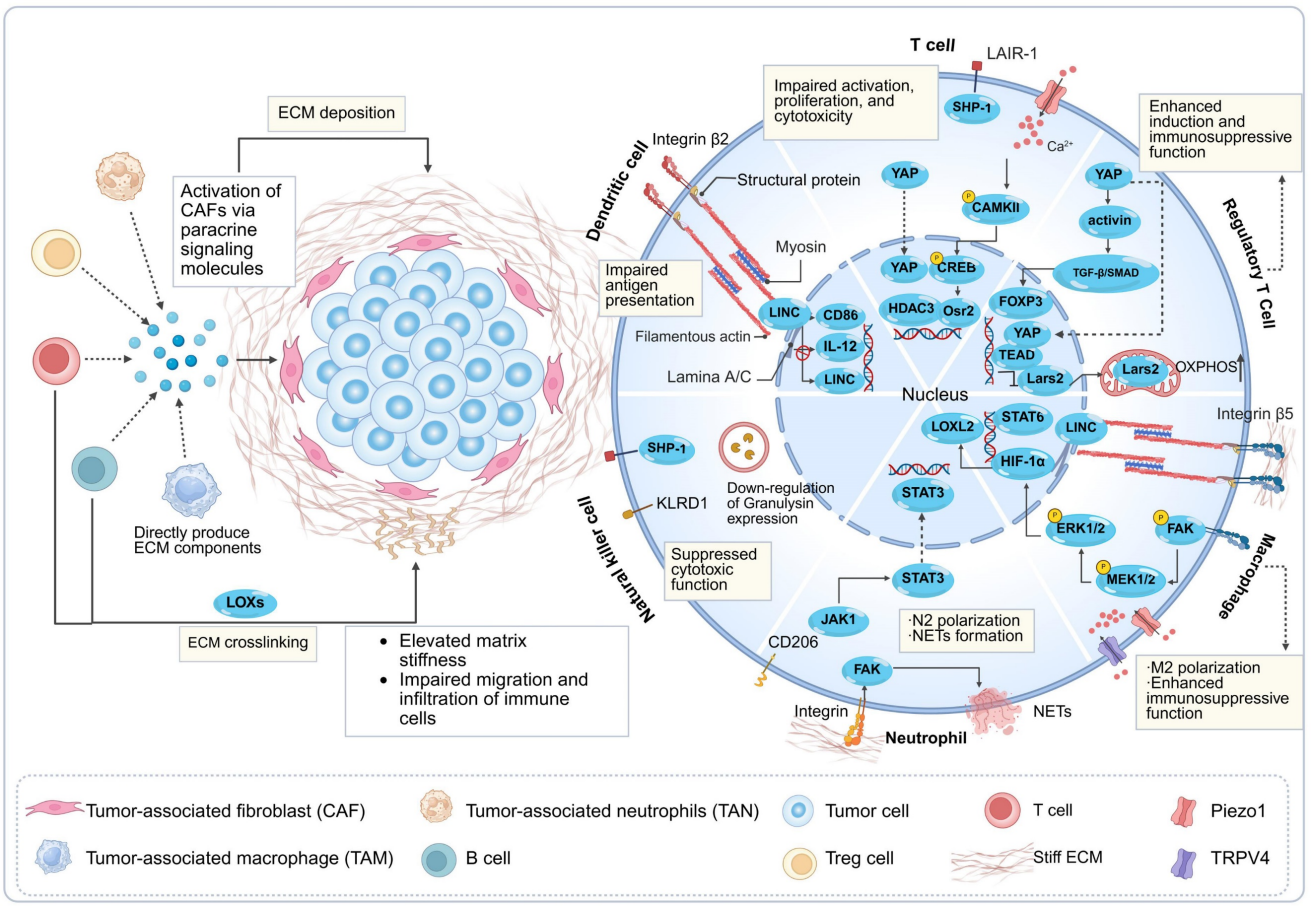
** Bidirectional regulation between matrix stiffness and immune cells. (A)** Immune cells promote matrix stiffening by directly secreting extracellular matrix (ECM) components and cross-linking enzymes (e.g., lysyl oxidase (LOX)), or by activating cancer-associated fibroblasts (CAFs) through paracrine signaling molecules such as transforming growth factor-β (TGF-β). These actions lead to excessive ECM deposition and enhanced cross-linking, resulting in elevated matrix stiffness that forms a physical barrier, thereby impeding immune cell infiltration and migration. **(B)** Conversely, increased matrix stiffness modulates immune cell function through mechanosensors (e.g., integrins, mechanosensitive ion channels) and downstream effectors (e.g., YAP/TAZ, JAK/STAT), ultimately impairing the cytotoxicity of effector cells (e.g., CD8⁺ T cells and natural killer cells), disrupting antigen presentation by dendritic cells, and enhancing the immunosuppressive activity of regulatory T cells. This mechano-immunological crosstalk fosters an immunosuppressive niche that facilitates tumor immune evasion.

Importantly, as key executors of antitumor immunity, immune cells actively participate in ECM remodeling by dynamically sensing and responding to changes in tumor matrix stiffness through mechanotransduction signaling pathways 30. Immune cells can directly or indirectly promote ECM deposition and crosslinking by secreting ECM-related proteins 31, collagen-modifying enzymes 12, and cytokines such as transforming growth factor-β (TGF-β) 32, thereby contributing to pathological matrix stiffening 33. Beyond forming a physical barrier that impedes immune infiltration, current evidence indicates that mechanostimuli mediated by elevated matrix stiffness activate cell membrane receptors and mechanosensors—including integrins, mechanosensitive ion channels, and cytoskeletal components 34-37. This biomechanical signaling cascade converts mechanical cues into biochemical responses, ultimately suppressing activation states, migratory capacities, and effector functions of key immune cells such as effector T cells, DCs, and Natural Killer (NK) cells, ultimately creating an immunosuppressive environment 38-40. In summary, a bidirectional regulation is formed between matrix stiffness and immune cells, continuously exacerbating the immunosuppressive state and ultimately jointly promoting tumor immune evasion 4, 41, 42 (**Fig. 1**).

Although several existing reviews have addressed the regulatory effects of matrix stiffness on immune cells 43, 44, the specific mechanisms by which matrix stiffness modulates diverse immune cell functions—such as migration, proliferation, differentiation, and secretory activity—remain inadequately elucidated. In addition, given the dynamic nature of matrix stiffness evolution, immune cells can actively remodel matrix mechanical properties to establish bidirectional crosstalk. However, this critical mechanism has been insufficiently emphasized in current research efforts.

In this review, we systematically dissect the key roles of immune cells in the development of elevated matrix stiffness in tumors and explore the biomechanical mechanisms through which stiffness modulates immune cell functions to drive immune evasion. On this basis, we propose an innovative theoretical framework: the matrix stiffness-immune cell bidirectional regulation axis, offering a biomechanical perspective for understanding immune evasion. Although stiffness-based metrics hold great promise for tumor diagnosis, staging, and prognostic stratification 10, 38, 45, their translation into therapeutic strategies remains limited. Therefore, we further evaluate the impact of matrix stiffness on immunotherapy efficacy, review recent clinical advances in targeting stiffness to enhance therapeutic responsiveness, and propose a combinatorial strategy integrating stiffness-targeting agents with immunotherapy. This approach aims to open new avenues for overcoming immunotherapy resistance and to provide actionable targets for clinical intervention.

## Main Text

### 1. Immune Cells Participate in Constructing the Hardened Fortress of the TME

The ECM constitutes a three-dimensional (3D) dynamic supramolecular network composed of collagen, elastin, proteoglycans, glycosaminoglycans, and functional glycoproteins (e.g., fibronectin, laminins), exhibiting distinctive physical, chemical, and biomechanical properties 46, 47. During tumor progression, aberrant deposition and excessive crosslinking of collagen and other ECM components serve as primary drivers of elevated matrix stiffness 17, 38, 48, 49. This process does not occur in isolation but is co-regulated by cellular components—such as tumor cells, immune cells, and CAFs—as well as acellular factors including the hypoxic microenvironment, collectively driving alterations in the mechanical properties of the TME 18, 20. Hypoxia, a hallmark of the TME, promotes excessive collagen synthesis and deposition by CAFs through hypoxia-inducible factor (HIF)-dependent mechanisms, while also inducing ECM-remodeling enzymes such as LOX to facilitate collagen cross-linking, ultimately leading to increased matrix stiffness 19, 50. For instance, hypoxia upregulates LOX expression via the HIF-1α-miR-142-3p axis; LOX then catalyzes the formation of rigid collagen networks, further enhancing matrix stiffness 19. This elevated stiffness, in turn, exacerbates intratumoral hypoxia 19, 50. Beyond ECM modulation, the hypoxic microenvironment recruits immuno-suppressive cells—including tumor-associated macrophages (TAMs) and tumor-associated neutrophils (TANs)—thereby fostering an immune-evasive milieu 50.

**Figure 2:**
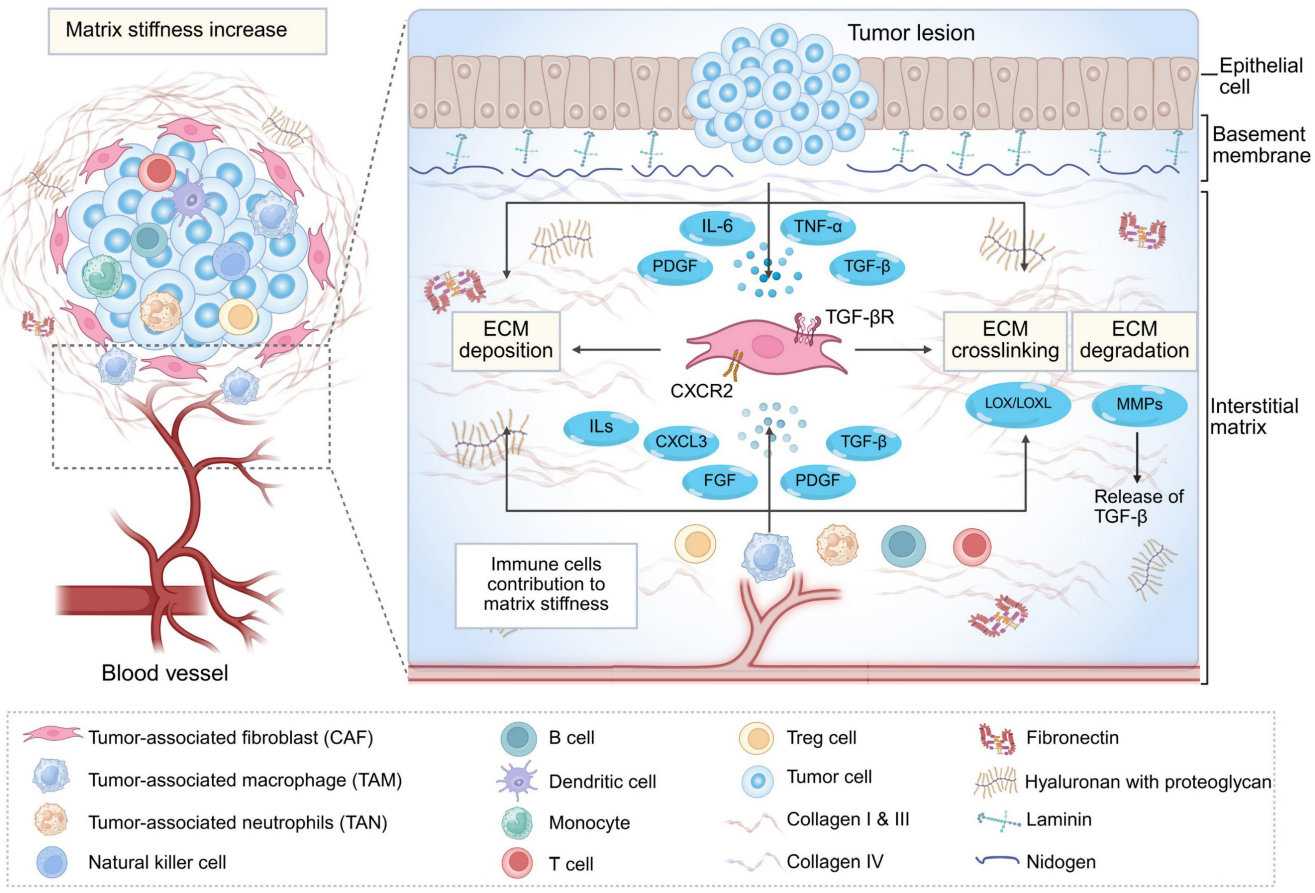
** The process of aberrantly increased tumor matrix stiffness.** The tumor extracellular matrix (ECM) is structurally categorized into the basement membrane and the interstitial stroma. The basement membrane comprises components such as laminin, type Ⅳ collagen, and nidogen, whereas the interstitial stroma is enriched with type Ⅰ/Ⅲ collagen, proteoglycans, and diverse glycoproteins, critically contributing to the high stiffness characteristic of the tumor stroma. Tumor cells and infiltrating immune cells, including tumor-associated macrophages (TAMs), not only directly secrete matrix proteins but also activate cancer-associated fibroblasts (CAFs) through the release of cytokines and chemokines. Activated CAFs, serving as the predominant source of interstitial ECM, extensively secrete and deposit ECM components. Lysyl oxidase (LOX) and lysyl oxidase-like (LOXL) family enzymes, secreted by TAMs, CAFs, and tumor cells, catalyze the crosslinking of ECM proteins. Concurrently, matrix metalloproteinases (MMPs) secreted by these same cell types degrade the ECM, thereby releasing latent transforming growth factor-β (TGF-β). Collectively, these processes drive the formation of a dense molecular network within the matrix, consequently elevating tumor matrix stiffness.

It is noteworthy that immune cells also play an important role in the dynamic regulation of the ECM 20, 31, 33, 51. They can not only directly secrete ECM components but also activate CAFs by producing cytokines and chemokines, thereby indirectly promoting ECM deposition 17, 33. Moreover, they can express collagen-modifying enzymes to regulate the spatial assembly process and stability of collagen 31, ultimately leading to an increase in matrix stiffness 33. Intriguingly, immune cells exhibit a dual role in ECM remodeling: while they facilitate matrix stiffening via the above mechanisms, they also secrete matrix metalloproteinases (MMPs) to degrade ECM components 31, 52-54. This seemingly contradictory functionality underscores the dynamic nature of immune-stromal crosstalk 33. Importantly, elevated matrix stiffness represents a dynamically regulated process throughout tumor progression and serves as a key biomechanical hallmark of aberrant ECM remodeling 18, 38 (**Fig. 2**).

#### 1.1 Immune cell involvement in ECM deposition

In the pathological remodeling process of tumor ECM, abnormal deposition of matrix proteins occurs, leading to changes in the density, structural organization, and porosity of the matrix, with a significant increase in matrix stiffness 13. Immune cells contribute to the elevated stiffness of the tumor matrix through two principal mechanisms that facilitate ECM deposition: firstly, via direct secretion of ECM components such as collagen, fibronectin, and osteopontin; and secondly, by indirectly promoting CAF-mediated matrix production through complex cytokine networks, including TGF-β and platelet-derived growth factor-B 31, 51, 55. Significantly, TAMs play a critical role in this process by engaging in a positive feedback loop with the progressively stiffening matrix. This reciprocal interaction further drives sustained ECM hardening 56, 57.

##### 1.1.1 Direct secretion of ECM-associated factors by immune cells

Recent studies reveal TAMs as active ECM remodelers that directly synthesize and secrete osteopontin, fibronectin, proteoglycans, and various collagen types, thereby influencing the mechanical properties and structural integrity of tumor tissue 31, 55. For instance, integrated transcriptomic and proteomic analyses in colorectal cancer models demonstrate that TAMs markedly upregulate gene expression and protein translation of type I (COL1A1/COL1A2), III (COL3A1), IV (COL6A1/COL6A3), and XIV (COL14A1) collagens. Depletion of TAMs results in widespread reduction of ECM proteins 55. Similarly, Peng *et al.* confirmed the pivotal role of TAMs in ECM deposition through single-cell RNA sequencing 58, an effect amplified by mechanochemical coupling. In 3D models simulating the high-stiffness microenvironment of triple-negative BC, mechanically stressed M2-like TAMs exhibit upregulated expression positively correlating with aberrant deposition of basement membrane heparan sulfate proteoglycan 2 (Perlecan/HSPG2). This deposition further elevates matrix stiffness, which in turn promotes Perlecan/HSPG2 expression in M2-like TAMs via nuclear factor kappa-light-chain-enhancer of activated B cells (NF-κB) pathway activation, establishing a stiffness-amplifying feedback loop 56. Thus, TAMs actively contribute to tumor matrix stiffening through direct collagen synthesis, with their secretory behavior mechanoregulated and engaged in reciprocal interactions with the mechanical microenvironment to perpetuate a vicious cycle.

Apart from TAMs, neutrophils have also been reported to actively contribute to ECM generation 59, 60. For instance, a specific subset of neutrophils identified in skin tissue directly participates in the structural and mechanical regulation of barrier tissues through de novo synthesis of ECM components 59. In models of myocardial infarction, neutrophils appear to promote ECM deposition in the ischemic heart via production of fibronectin and fibrinogen 60. However, these findings have not yet been fully validated within the TME, highlighting a significant gap in current understanding. Nonetheless, whether other immune cells directly secrete ECM proteins remains largely unexplored, representing a novel avenue for future research.

##### 1.1.2 Immune cell-mediated indirect promotion of ECM deposition via CAF activation

CAFs, the most abundant stromal cell population in the TME, serve as primary orchestrators of ECM remodeling and stiffness elevation. They achieve this through excessive production of structural ECM proteins (e.g., collagens type I, III, IV, V, laminin, fibronectin) and crosslinking enzymes such as LOXs 26, 61. Immune cells—including TAMs, T cells, Tregs, TANs, and B cells—secrete cytokines such as CXC-motif chemokines, TGF-β, fibroblast growth factors, interleukins (ILs), and platelet-derived growth factors, which potently augment CAFs activity. Specifically, TGF-β activates CAFs via SMAD-dependent and -independent pathways, enhancing synthesis, secretion, and deposition of matrix proteins to promote matrix stiffening 20, 32, 51, 62, 63. Acerbi *et al.* reported that in BC, TGF-β signaling intensity, matrix stiffness, and infiltrating activated TAMs exhibit positive mutual correlation 48. High matrix stiffness potentiates SMAD signaling downstream of TGF-β and stimulates TAMs to produce elevated levels of active TGF-β1. TGF-β1 then induces CAFs to synthesize fibrillar collagens and collagen crosslinking enzymes, driving stiffness escalation 57. In pancreatic ductal adenocarcinoma (PDAC), TAM-derived C-X-C motif chemokine ligand 3 engages C-X-C chemokine receptor 2 on CAFs to induce myofibroblastic transdifferentiation, accelerating collagen type Ⅲ deposition 64.

In addition to TAMs, B cells derived from PDAC patients secrete the pro-fibrotic mediator platelet-derived growth factor-B, which directly stimulates collagen production by fibroblasts 51. Additionally, neutrophil extracellular traps (NETs)—web-like structures composed of DNA filaments decorated with histones and granule proteins released by TANs 65—enhance the migratory capacity of hepatic stellate cells, promoting their recruitment to metastatic liver lesions and subsequent transformation into CAFs, thereby indirectly facilitating ECM synthesis and deposition 66. This immune cell-initiated CAF activation, culminating in excessive ECM deposition and aberrant crosslinking, constitutes an important indirect mechanism of tumor matrix stiffening.

#### 1.2 Immune cell involvement in ECM modification

The biomechanical properties of the ECM are governed by post-translational modifications—including hydroxylation, glycosylation, and crosslinking—which collectively contribute to its structural integrity, stability, and matrix stiffness 13, 17, 67. Hydroxylation, catalyzed by prolyl 4-hydroxylases (P4Hs) and lysyl hydroxylases (PLODs) within the endoplasmic reticulum, represents an initial critical step that facilitates correct collagen folding into stable triple helices and serves as a key precursor for covalent cross-linking 68, 69. P4H isoforms are frequently overexpressed in tumors and promote ECM remodeling, tumor cell invasion, and metastasis through HIF-1α-dependent expression 70. Following hydroxylation, glycosylation of hydroxylysine residues further modulates collagen maturation and enhances its resistance to proteolytic degradation, thereby prolonging ECM persistence 17, 71. After secretion and proteolytic processing, collagen fibers undergo cross-linking primarily mediated by LOXs, which oxidize lysine and hydroxylysine residues to generate reactive aldehydes that facilitate intermolecular covalent bond formation. In the tumor context, aberrant LOXs activity drives pathological stiffening through excessive cross-linking, severely compromising matrix deformability and serving as a major driver of stromal hardening 67, 72.

Beyond the roles of tumor cells and CAFs, immune cells also actively regulate post-translational modification of the ECM 31, 73. TAMs directly overexpress collagen-modifying enzymes—including PLOD1/3, P4HA1, procollagen C-endopeptidase enhancer, and transglutaminase 1/2—to control collagen stability and spatial assembly, thereby enhancing crosslinking 55, 57, 73, 74. Maller *et al.* demonstrated that TAMs directly drive fibrotic progression in invasive breast tumors by stimulating collagen crosslinking and matrix hardening 73. Similarly, Xing *et al.* reported that high expression of collagen type I/LOX in HCC correlates positively with M2-like TAMs and lysyl oxidase-like 2 (LOXL2) levels. Their work further linked matrix stiffness to LOXL2 upregulation via the integrin β5-focal adhesion kinase (FAK)-mitogen-activated protein kinase kinase 1 and 2 (MEK1/2)-extracellular signal-regulated kinase 1/2 (ERK1/2) signaling axis 12. Other immune populations also contribute to ECM remodeling: B cells in PDAC express LOXL2 51, while CD8⁺ T cells serve as a major source of LOX following chemotherapy in BC, mediating treatment-induced ECM remodeling in the lung 75. These findings systematically illuminate how immune cells promote tumor stiffness through enzymatic ECM modification and cross-linking, providing a mechanistic rationale for therapeutic strategies targeting immune-stromal crosstalk.

#### 1.3 Immune cells participate in ECM degradation

The ECM undergoes continuous remodeling through a dynamic process of constant synthesis and degradation, wherein the degradation of ECM components facilitates the replacement of the normal ECM architecture with a tumor-derived ECM matrix 17. MMPs represent the principal enzyme family responsible for degrading collagen and other structural proteins within the ECM 76. Based on substrate specificity and domain organization, MMPs are classified into several types, including collagenases (e.g., MMP-1, MMP-8, MMP-13), gelatinases (e.g., MMP-2, MMP-9), and membrane-type MMPs (MT-MMPs). Among these, MMP-2 and MMP-9 are the most extensively studied MMPs, as they facilitate tumor invasion and metastasis by degrading the ECM and basement membrane, thereby creating physical pathways for tumor dissemination 77. Within the TME, both myeloid and lymphoid cells secrete MMPs; nonetheless, the types, levels, and functions of MMPs secreted exhibit cell-type specificity 31, 52-54. Myeloid cells are significant contributors to MMP production. For instance, TANs secrete MMP-2, MMP-9, and MMP-8, whereas TAMs release MMP-2, MMP-9, and MMP-12, among others 31, 52, 53, 78. Studies have demonstrated that MMPs secreted by these cells promote ECM degradation, angiogenesis, and tumor metastasis 31, 52, 53, 78. In contrast, although T cells are capable of transcribing mRNAs for proteases such as MMP-9, MT1-MMP, and MT4-MMP, they exhibit markedly low cell surface protein expression 54. Within the TME, MMP expression in T cells is primarily associated with facilitating migration and infiltration rather than broad ECM degradation 79. These functional disparities underscore the diverse regulatory roles of immune cell-specific MMP expression in ECM remodeling and tumor progression.

MMP-mediated ECM degradation is a critical driver of tumor cell migration and invasion 17. Although extensive proteolysis may transiently reduce local matrix stiffness 77 and could theoretically enhance immune cell infiltration, it concurrently liberates latent TGF-β and other bioactive fragments embedded within the matrix. The released TGF-β not only directly suppresses immune effector functions but also activates CAFs, prompting them to synthesize and deposit new matrix components 80-82. This process ultimately leads to the progressive remodeling and replacement of the native ECM with a tumor-derived, pathologically altered stroma 42. Interestingly, myeloid cells can also secrete tissue inhibitor of metalloproteinases-1 (TIMP-1), which counteracts MMP activity 36, 83. The dynamic balance between MMPs and TIMPs is regulated by tumor stage and microenvironmental signals 84. Thus, the consequences of ECM degradation are highly context-dependent, shaped by the spatiotemporal patterns of protease activity, the composition of degradation products, and ongoing crosstalk with stromal cells.

### 2. The Tumor Immune Evasion Greenhouse Nurtured by Matrix Stiffness

Elevated matrix stiffness acts not only as a biomechanical driver of tumor malignancy but also as a key regulatory factor that induces an immunosuppressive TME and promotes immune evasion 7, 8. Immune cells actively contribute to ECM deposition and cross-linking 55. The resulting elevated matrix stiffness reciprocally modulates immune cell activation and effector functions through mechanotransduction pathways. This bidirectional "matrix stiffness-immune cell" crosstalk redefines the intrinsic logic of tumor stromal evolution and offers novel perspectives for understanding immune evasion and developing targeted therapeutic strategies.

First, densely crosslinked rigid matrices form physical barriers that impede the recruitment of effector immune cells, allowing tumor cells to escape immunosurveillance 23, 40. Second, immune cells actively perceive and respond to stiffness variations through mechanotransduction 30, primarily mediated by membrane receptors and mechanosensors that globally activate immunosuppressive networks 35, 43. For instance, mechanical stimuli induce conformational changes in adhesion complexes (e.g., integrin clusters), recruiting adaptor proteins to activate signaling cascades such as the phosphatidylinositol 3-kinase (PI3K)/protein kinase B (AKT) pathway. These forces propagate via the cytoskeleton to drive nuclear translocation of mechanosensitive transcription factors including Yes-associated protein (YAP)/transcriptional coactivator with PDZ-binding motif (TAZ). Meanwhile, mechanosensitive ion channels rapidly transduce physical cues into biochemical responses via secondary messengers (e.g., Ca²⁺ influx). Collectively, these mechanotransduction events orchestrate protein refolding, cytoskeletal reorganization, and nuclear shuttling of transcriptional regulators—ultimately modulating immune cell activation, differentiation, and effector functions 35, 85. As a result, the cytotoxicity of T cells and NK cells is impaired, DCs exhibit impaired antigen presentation, and immunosuppressive cells such as TAMs and Tregs accumulate and activate 32, 43. In summary, matrix stiffness impedes immune cell function via a dual mechanism involving physical barriers and mechanical signaling. This results in compromised antitumor immune responses and fosters an environment conducive to tumor immune evasion 4, 41. Herein, we primarily focus on discussing the specific mechanisms through which matrix stiffness influences the function of immune cells.

#### 2.1 Matrix stiffness modulates macrophage polarization and immunoregulatory functions

**Figure 3:**
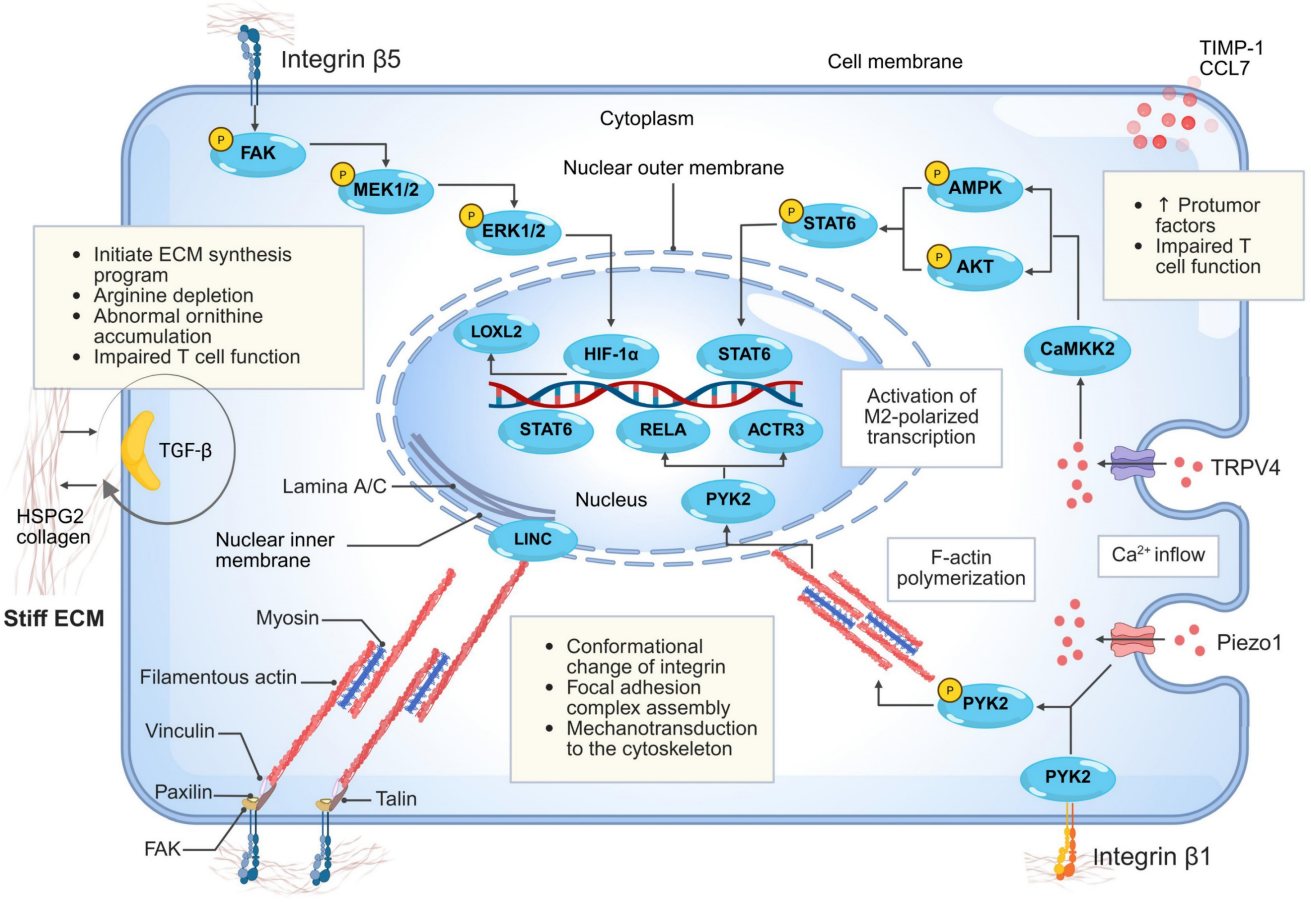
** Mechanisms by which elevated matrix stiffness modulates macrophage polarization and immunoregulatory functions.** Increased stiffness transmits signals via the integrin β5-cytoskeleton axis to enhance chromatin accessibility at M2 gene promoter regions, initiating M2-polarizing transcriptional programs. In hepatocellular carcinoma, stiffness promotes M2 polarization through the integrin β5-focal adhesion kinase (FAK)-mitogen-activated protein kinase kinase 1 and 2 (MEK1/2)-extracellular signal-regulated kinase 1/2 (ERK1/2)-hypoxia-inducible factor 1-α (HIF-1α)-lysyl oxidase-like 2 (LOXL2) signaling cascade. Concurrently, activation of mechanosensitive channels Piezo1 and TRPV4 induces Ca²⁺ influx, leading to Ca²⁺/calmodulin-dependent protein kinase kinase 2 (CaMKK2) activation and subsequent STAT6 nuclear translocation. In a complementary pathway, Piezo1-mediated Ca²⁺ signaling synergizes with the integrin β1-F-actin axis to activate proline-rich tyrosine kinase 2 (PYK2), which translocates to the nucleus and binds promoters of RELA and Arp2/3 complex subunits (e.g., ACTR3), further promoting M2 differentiation. Furthermore, stiffness-induced M2-like TAMs upregulate heparan sulfate proteoglycan 2 (HSPG2), whose aberrant deposition creates a positive feedback loop that further elevates matrix stiffness. Additionally, high stiffness stimulates TAMs to secrete pro-tumor factors (e.g., TIMP-1, CCL7), induces autocrine transforming growth factor-β (TGF-β)-SMAD signaling to promote collagen synthesis, and drives metabolic reprogramming via the TGF-β-arginase-1 axis, resulting in arginine depletion and ornithine accumulation that collectively impair CD8⁺ T cell function.

Macrophages exhibit high phenotypic and functional plasticity. Beyond presenting exogenous antigens, they secrete cytokines and growth factors to orchestrate immune responses. Within tumors, these cells are defined as TAMs. TME-derived signals drive TAM polarization toward classically activated (M1) or alternatively activated (M2) phenotypes, with M2-like TAMs exhibiting anti-inflammatory and immunosuppressive functions 86, 87. Accumulating evidence indicates that the biophysical properties of the ECM—notably matrix stiffness—exert significant regulatory effects on macrophage polarization and function 11. Through multiple mechanotransduction signaling pathways, elevated matrix stiffness promotes M2-like polarization of macrophages and enhances their immunosuppressive activity. These alterations ultimately contribute to the impairment of CD8⁺ T cell function and foster tumor immune evasion 35, 42, 43, 57 (**Fig. 3**).

##### 2.1.1 Matrix stiffness promotes M2 polarization

The process by which elevated tumor matrix stiffness drives macrophage polarization toward an M2 phenotype involves multiple interconnected layers, including mechanoreceptor activation, cytoskeletal-nuclear coupling, signaling cascades, and transcriptional reprogramming 11, 36, 56, 88, 89. This regulation is initiated by mechanosensing and signal transduction. Integrins—core mediators of cell-ECM adhesion—play pivotal roles in stiffness-induced M2 polarization 11, 12, 36. When macrophages adhere to high-stiffness polyacrylamide (PA) gels, conformational changes in integrin family receptors (e.g., integrin β5) lead to the recruitment of adaptor proteins such as talin and paxillin, followed by the assembly and activation of FAK, collectively forming focal adhesion complexes. This complex transmits mechanical signals to the cytoskeleton via myosin-assembled stress fibers 36, 85, 90. The forces are subsequently relayed through the linker of nucleoskeleton and cytoskeleton complex into the nucleus, leading to nuclear flattening and upregulation of lamin A/C expression. This enhances chromatin accessibility at M2-associated gene promoters, facilitating binding of key transcription factors like signal transducer and activator of transcription 6 (STAT6) to activate pro-M2 transcriptional programs 36, 90. Intriguingly, this mechanical-transcriptional regulation synergizes with biochemical signals such as IL-4/IL-13, and cooperatively drives the alternative M2 activation program 36, 90. In HCC, elevated matrix stiffness activates the integrin β5-FAK-MEK1/2-ERK1/2 signaling axis, leading to upregulation of HIF-1α expression. This mechanotransduction pathway subsequently promotes the expression of M2 macrophage polarization markers and LOXL2, collectively enhancing M2-polarization of macrophages in the TME 12.

In addition to the integrin-cytoskeleton-nucleus axis, the activation of mechanosensitive ion channels provides another critical transduction pathway for matrix stiffness-mediated macrophage polarization 11, 89. Studies using 3D stiffened hydrogel models have demonstrated that elevated matrix stiffness activates Piezo type mechanosensitive ion channel component 1 (Piezo1) and transient receptor potential vanilloid 4 (TRPV4) mechanosensitive channels in macrophages, inducing extracellular Ca²⁺ influx 34, 89, 91. Notably, Piezo1 responds to high-intensity mechanical stimuli by generating transient yet high-amplitude Ca²⁺ signals, whereas TRPV4 is sensitive to low-intensity, sustained mechanical cues and produces prolonged Ca²⁺ oscillations. TRPV4 often acts downstream of upstream mechanosensors such as Piezo1, primarily functioning as a Ca²⁺ signal amplifier 11, 92. The resulting elevation in intracellular Ca²⁺ concentrations further activates calcium/calmodulin-dependent protein kinase kinase 2 (CaMKK2) 93. CaMKK2 phosphorylates downstream effectors including AMP-activated protein kinase (AMPK) and AKT, ultimately promoting STAT6 nuclear translocation and M2-associated gene transcription 89. Intriguingly, Xie *et al.* revealed in a PDAC model that elevated matrix stiffness cooperatively activates both Piezo1-mediated Ca²⁺ influx and the integrin β1-filamentous actin signaling axis, which together induce phosphorylation of the proline-rich tyrosine kinase 2 (PYK2) in the cytoplasm. Activated PYK2 not only amplifies mechanical signals by modulating cytoskeletal reorganization but also translocates into the nucleus, where it directly binds to the promoter regions of genes such as v-rel reticuloendotheliosis viral oncogene homolog A and subunits of the Arp2/3 complex, driving their transcription and thereby promoting monocyte differentiation into M2-like macrophages 11.

However, current research findings regarding the relationship between matrix stiffness and macrophage polarization remain conflicting 94, 95, primarily due to technical variations in experimental systems and the inherent biological complexity of the TME. Specifically, many investigations rely on 2D substrates, which fail to recapitulate the architectural and mechanical complexity of native tumor ECM. Furthermore, the definition of "stiffness" varies considerably across studies, lacking standardized quantitative ranges. Substantial differences in cellular models—ranging from transformed cell lines such as THP-1 human monocytes to primary bone marrow-derived macrophages—also contribute to divergent mechanoresponsive behaviors and polarization outcomes 89.

Taken together, matrix stiffness biases macrophage polarization toward the M2 phenotype through multiple mechanosignaling pathways and synergistic interactions with biochemical signals. The polarized M2 macrophages subsequently establish an immunosuppressive microenvironment by secreting inhibitory cytokines, impairing the function of effector T cells and NK cells, and recruiting immunosuppressive cells such as Tregs. Collectively, these mechanisms promote immune evasion and tumor growth 86.

##### 2.1.2 Matrix stiffness promotes the migration and infiltration of M2-like TAMs

The spatial distribution of macrophage subsets within the TME is influenced by their phenotype-specific migratory and adhesive behaviors. Elevated matrix stiffness modulates the expression of adhesion- and migration-related molecules in TAMs, potentially favoring the infiltration of M2-like TAMs into stiffer matrix regions 12, 42, 96, 97. This provides a plausible mechanistic explanation for the positive correlation between increased matrix stiffness and a higher proportion of M2-like TAMs observed in solid tumors such as head and neck squamous cell carcinoma 36, 88. Such stiffness-guided migration enables M2-like TAMs to penetrate deeply into tumor parenchyma, engage in close interactions with cancer cells, and amplify their immunosuppressive functions 42.

Integrin-mediated adhesion represents a core mechanism governing the 3D migration of macrophages, a process highly dependent on integrin expression levels 97. Within the TME, elevated matrix stiffness acts as a critical biomechanical cue that coordinates integrin-dependent migration by regulating integrin activation, adhesion complex dynamics, cytoskeletal reorganization, and downstream signaling pathways, thereby determining migratory mode and efficiency 96. Compared to M1-like TAMs, M2-like TAMs exhibit higher expression of integrin β5 12 and β3 98. Both integrins recognize the RGD motif and synergy sites within fibronectin—a component enriched in stiffened tumor stroma 6, 14—and couple extracellular adhesion to intracellular mechanical functions via linkage to the actin cytoskeleton through cytoplasmic adaptor proteins 99. Increased matrix stiffness facilitates force transmission, promoting the maturation of nascent adhesions into focal adhesions and fibrillar adhesions. Integrins, particularly αvβ3, exhibit enhanced activation in stiffer regions, leading to recruitment of talin and paxillin, which stabilize adhesion complexes and direct durotactic migration 96. Notably, high stiffness upregulates integrin β5 expression in M2-like TAMs 12, further promoting their mechanotactic migration 96. Concurrently, elevated tumor stiffness enhances the secretion of MMPs in M2-like TAMs 100. MMPs not only degrade ECM components 31 but also cleave fibronectin to expose cryptic RGD sites, thereby amplifying matrix remodeling and invasive capacity 96. Together, these mechanisms drive the preferential accumulation of M2-like TAMs in stiffened areas of the tumor ECM, where their abundance correlates positively with matrix stiffness 42, thereby facilitating immunosuppression and promoting tumor immune evasion.

##### 2.1.3 Matrix stiffness enhances TAM-mediated immunosuppression

Matrix stiffness not only directly polarizes macrophages toward the immunosuppressive M2 phenotype but also augments their protumor factor secretion and exacerbates metabolic dysregulation in the TME, collectively impairing antitumor T cell responses 57, 101. Clinical analyses reveal that elevated matrix stiffness in head and neck squamous cell carcinoma correlates with upregulated secretion of pro-tumor factors (e.g., TIMP-1, C-C motif chemokine ligand 7, and angiopoietinrelated protein 3) by M2-like TAMs 36, 88. What's more, in metastatic ovarian cancer, elastic moduli can be 100-fold higher than in healthy omentum; such stiff matrices induce M2-like TAMs to overexpress TGFBI, suppressing CD8^+^ T cell function 101.

Macrophages infiltrating the high-stiffness TME exhibit enhanced immunosuppressive activity. Their conditioned medium directly suppresses T-cell proliferation and impairs the secretion of effector molecules such as interferon-γ (IFN-γ) 36, 102, while autocrine activation of the TGF-β signaling pathway exacerbates metabolic dysregulation in the BC TME 57. For example, in BC models, matrix stiffness remodels TAM function through a dual mechanism: on one hand, it induces autocrine TGF-β release from TAMs, activating the TGF-β/SMAD pathway to promote collagen synthesis; on the other hand, it drives metabolic reprogramming via the TGF-β-arginase-1 axis, enhancing arginine uptake and proline synthesis. This leads to excessive ornithine production and release into the TME, resulting in arginine depletion and ornithine accumulation 57. Arginine depletion impairs adenosine triphosphate (ATP) synthesis and effector molecule production in T cells 103, while ornithine competitively inhibits solute carrier family 7 member A transporter-mediated arginine uptake, disrupting metabolic homeostasis in T cells—manifested as elevated oxidized glutathione and reduced ATP levels. These changes collectively inhibit CD8⁺ T-cell proliferation, activation, and tumor-killing capacity, and diminish their response to immune checkpoint blockade (ICB) therapy 57.

In aggregate, these findings elucidate the mechanobiological mechanisms by which stiffness amplifies TAM-driven immunosuppression to facilitate immune evasion. The following section details stiffness-mediated biomechanical regulation of T cell function.

#### 2.2 Matrix stiffness suppresses T cell activation, migration, and cytotoxic function

**Figure 4:**
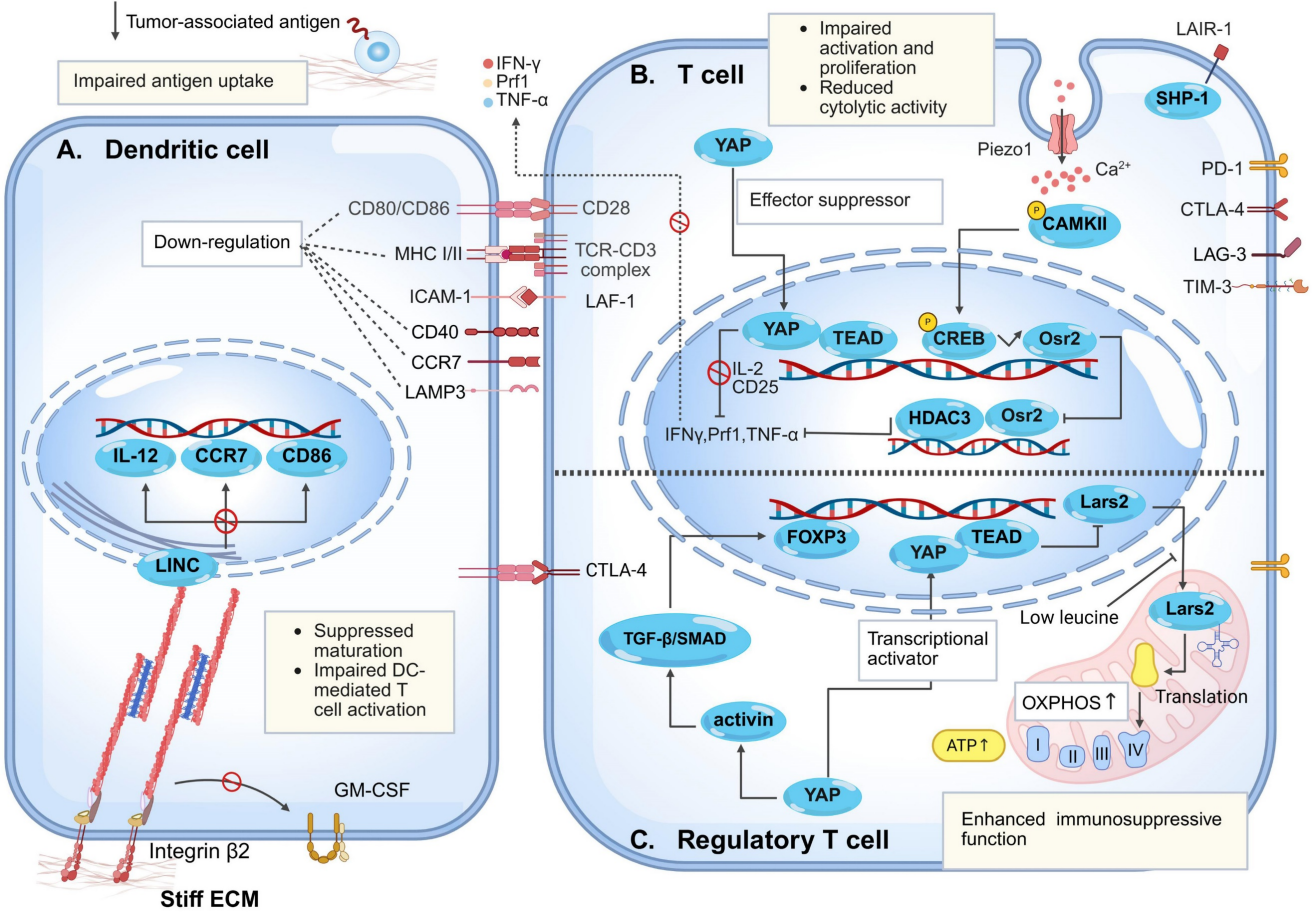
** Mechanism of high matrix stiffness in regulating dendritic cells, T cells, and regulatory T cells. (A)** Elevated matrix stiffness impairs DCs antigen capture capacity by reducing tumor immunogenicity and creating a physical barrier. Simultaneously, increased stiffness reinforces integrin β2-cytoskeleton linkage, leading to negative regulation of granulocyte-macrophage colony-stimulating factor (GM-CSF) signaling and triggering Lamin A/C-mediated chromatin condensation. This directly suppresses the transcription of maturation-related genes, including CD86, interleukin-12 (IL-12), and CC-chemokine receptor 7 (CCR7), resulting in arrested DC maturation. Furthermore, via β2 integrin signaling, high stiffness downregulates the expression of co-stimulatory molecules (CD80/CD86) and CCR7 on mature DCs, thereby further inhibiting T cell activation. **(B)** Within T cell-related regulatory mechanisms, elevated matrix stiffness promotes nuclear translocation of Yes-associated protein (YAP), which forms a complex with TEA domain transcription factor (TEAD) to suppress the expression of T cell activation-related genes (e.g., IL-2, CD25) and impair proliferative capacity. Concurrently, it downregulates the expression of effector molecules such as interferon-γ (IFN-γ) and tumor necrosis factor-α (TNF-α), thereby inhibiting CD8⁺ T cell cytotoxicity. On the other hand, high stiffness upregulates the expression of leukocyte-associated immunoglobulin-like receptor 1 (LAIR1), which recruits Src homology 2 domain-containing protein tyrosine phosphatase-1 (SHP-1) and contributes to T cell exhaustion. Additionally, Piezo1 activation via the Ca²⁺-calcium/calmodulin-dependent protein kinase Ⅱ (CaMKⅡ)-cAMP response element-binding protein (CREB) axis upregulates odd-skipped related transcription factor 2 (Osr2), which recruits histone deacetylase 3 (HDAC3) to reduce cytotoxic gene histone acetylation, suppressing T cell function. **(C)** Elevated stiffness drives regulatory T cell (Treg) differentiation/maintenance via dual YAP mechanisms: enhancing mitochondrial oxidative phosphorylation (OXPHOS) and activating transforming growth factor-β (TGF-β)/SMAD-forkhead box P3 (FOXP3) signaling. These processes reinforce Treg immunosuppression, facilitating tumor immune evasion.

An effective anti-tumor immune response relies on the complete activation and cascade amplification of the "cancer-immunity cycle." During this process, DCs initiate T cell responses by presenting tumor antigens via major histocompatibility complex (MHC) Ⅰ/Ⅱ, followed by activated effector T cells migration to tumor sites for recognition and killing of tumor cells 104. Nonetheless, abnormally elevated matrix stiffness in tumor tissues prevents effective activation of this cycle. Acting as a regulator of adaptive immune cell behavior, high matrix stiffness suppresses T cells activation and proliferation, impairs their migratory capacity, and reduces tumor infiltration efficiency. More importantly, high matrix stiffness induces T cell exhaustion, significantly impairs their effector functions 35, 37, 105, and ultimately leads to tumor immune evasion and triggers clinical treatment resistance (**Fig. 4**).

##### 2.2.1 Matrix stiffness inhibits T cell activation and proliferation

Elevated matrix stiffness suppresses T cell activation and proliferation through multiple mechanisms, including disruption of immune synapse (IS) formation and regulation of the YAP signaling pathway 105, 106. IS formation represents a critical step in T cell activation, with its structure centered on the T cell receptor (TCR)-MHC-peptide complex, surrounded by adhesion molecules to form a "bullseye" architecture 107. Within the physiological stiffness range (approximately 5-20 kPa), the counterforce provided by the matrix enables TCR-pMHC bonds to sustain and maintain appropriate levels of tension, thereby stabilizing microclusters, prolonging their lifetime, and amplifying downstream signaling. Conversely, excessively high matrix stiffness may impose supraphysiological tension, leading to premature bond dissociation and disruption of microcluster stability—unless compensatory mechanical support is provided by integrins such as lymphocyte function-associated antigen 1 to preserve synaptic structure and function 108, 109. Consistent with this, Jin *et al.* utilized elastic micropillar arrays to demonstrate that elevated substrate stiffness delays the translocation of the microtubule-organizing center to the IS, ultimately impairing synapse formation and T-cell activation 106.

In addition to synaptic regulation, studies utilizing 3D collagen-based matrices that mimic the features of solid tumor ECM have revealed that high matrix stiffness activates mechanosensing pathways, leading to YAP nuclear translocation and complex formation with TEA domain transcription factor (TEAD). This suppresses the expression of T-cell activation-related genes (e.g., IL-2, CD25) and impairs their proliferative capacity 105. Furthermore, investigations based on 3D *in vitro* models have shown that under high-stiffness conditions, CD8⁺ T-cell viability is more susceptible to impairment than that of CD4⁺ T cells, resulting in an altered CD4⁺/CD8⁺ ratio and reduced cytotoxic output 39. Importantly, T-cell proliferation exhibits a biphasic response to substrate stiffness, with the highest fold increase in cell numbers observed on 25 kPa PA gels 110. This may be attributed to the release of YAP from the cytoplasm and its subsequent nuclear import, which prevents its binding to IQ motif-containing GTPase-activating protein 1, thereby permitting nuclear translocation of nuclear factor of activated T-cells 1 and enhancing glycolytic and amino acid metabolic pathways in T cells to support proliferation 111. However, as stiffness increases beyond this point, T-cell proliferation demonstrates a declining trend 110.

It should be noted that most current studies are conducted on planar surfaces, whereas cell-matrix interfaces exhibit more complex spatial topologies; thus, some conclusions may differ from those derived from planar gel systems 106. Collectively, elevated matrix stiffness disrupts immune synapse formation, inhibits YAP/TEAD-dependent transcriptional activation, and ultimately causes defects in T-cell activation and proliferation. These effects compromise both the initial recognition of tumor antigens and the expansion of immune responses, thereby collectively impairing the anti-tumor functionality of T cells.

##### 2.2.2 Matrix stiffness restricts T cell infiltration and migration

Effective immune surveillance requires the precise localization and motility of T cells within tumors. For cytotoxic function, infiltrating T cells must navigate between and interact with tumor cells 112.

Nonetheless, in many tumor types, the desmoplastic reaction occurring in the TME is directly associated with increased matrix stiffness 22 and leads to the formation of dense connective tissue that encapsulates tumor nests 21, 27, 113. The infiltration and migratory capacity of T cells are severely impaired due to physical confinement 21, 114. Consequently, T cells are sparsely distributed within tumor nests and are instead predominantly enriched in the surrounding stromal regions 115. This restricted spatial distribution and heterogeneous clustering represent a key mechanism of immune evasion. Significantly, reducing matrix stiffness through collagenase treatment has been shown to enhance T cell-tumor cell contact and thereby improve anti-tumor immune responses 114-116.

Unlike adherent cells, T lymphocytes do not form substantial attachments to the ECM, instead transmitting frictional forces through retrograde flow of the actin cytoskeleton. This enables them to employ conserved migratory strategies across tissues with vastly different compositions 117. Consequently, matrix barriers exhibit comparable inhibitory effects on T cell migration across diverse tumor types. In ovarian and lung cancer tissues, T cells primarily accumulate in stromal regions and display marked migratory slowness 114, 115. In PADC, collagen-rich stroma traps infiltrating T cells, with activated T cells migrating efficiently in low-density collagen but suppressed in high-density environments 118. Mechanistic studies reveal that in loose fibronectin/collagen regions, T cells undergo rapid β1/β2-integrin-independent migration via chemokine receptor-mediated chemotaxis. Conversely, in dense matrices, single-cell migration capacity inversely correlates with collagen density and stiffness, culminating in peripheral trapping and failed infiltration 23.

Intriguingly, studies utilizing polyethylene glycol-fibrinogen hydrogels to mimic the stiffness of breast tumors have demonstrated that elevated matrix stiffness can also induce nuclear enlargement in T cells through membrane mechanocompression, thereby impairing their infiltrative capacity 39. This multifactorial regulatory network ultimately dictates T-cell distribution and functional states within tumors, thereby determining overall antitumor immune efficacy 116.

##### 2.2.3 Matrix stiffness suppresses T cell-mediated immune killing

Besides acting as a physical barrier, elevated matrix stiffness reshapes T cell function across multiple dimensions—including receptor signaling 119, ion channels 120, and transcriptional regulation 121, 122—through activation of multiple mechanosensitive pathways. This process induces T cell exhaustion, suppresses cytotoxic activity, and ultimately constitutes the core mechanical regulatory network driving tumor immune evasion 39, 40. In tumors with abundant collagen deposition (e.g., lung cancer), expression of the collagen receptor leukocyte-associated immunoglobulin-like receptor-1 (LAIR1) is significantly upregulated. LAIR1 recruits Src homology 2 domain-containing tyrosine phosphatase-1 (SHP-1) via its intracellular domain, directly inducing T cell exhaustion and rendering programmed cell death protein 1 (PD-1)/programmed death-ligand 1 (PD-L1) blockade therapy ineffective 119.

Concomitantly, activation of the Piezo1 in T cells represents another critical regulatory axis. Specifically, Piezo1 is highly expressed in activated CD8⁺ T cells and functions as a negative regulator of cytotoxicity. Inhibition of Piezo1 activity enhances T-cell traction forces and augments their tumor-killing capacity 120. Nonetheless, under conditions of high tumor matrix stiffness, Piezo1 activation upregulates odd-skipped related transcription factor 2 (Osr2) through the Ca²⁺-Ca²⁺/calmodulin-dependent protein kinase Ⅱ-cAMP response element-binding protein axis. Osr2 recruits histone deacetylase 3 to suppress cytotoxic gene transcription, impairing T cells function. Osr2 deletion reduces exhaustion, enhances antitumor responses, and improves chimeric antigen receptor T cell therapy (CAR-T) efficacy in solid tumors 37. Furthermore, *in vitro* models have confirmed that elevated matrix stiffness promotes nuclear translocation of YAP in effector T cells 111, 123. As a suppressor of T cells effector responses, enhanced YAP transcriptional activity directly impairs the cytotoxic function of CD8⁺ T cells, manifesting as downregulated expression of effector molecules such as IFN-γ and tumor necrosis factor-α (TNF-α) 105, 121.

Significantly, in many cancers, tumor-intrinsic alterations preferentially recruit and activate Tregs rather than effector T cells 124, 125. Accumulation of immunosuppressive Tregs is a key immune evasion strategy 126. Unlike immune effector cells, elevated matrix stiffness within tumors promotes cancer immune evasion by activating the mechanosensitive protein YAP, which induces the differentiation of Tregs, enhances their oxidative phosphorylation (OXPHOS) metabolism, and strengthens their immunosuppressive functions 122, 127, 128.

A 2D study utilizing PA hydrogels demonstrated that the differentiation efficiency of human CD4⁺CD25⁺ T cells into Tregs correlates positively with matrix Young's modulus, and Tregs display significant upregulation of mitochondrial genes associated with OXPHOS 127. Elevated OXPHOS rates not only facilitate Tregs induction but also sustain their anti-inflammatory phenotype and suppressive function 129. Mechanistically, employing both *in vivo* and *in vitro* tumor models, Ni *et al.* demonstrated that matrix stiffness-activated YAP plays a central regulatory role in the differentiation and functional maintenance of tumor-infiltrating Tregs (TI-Tregs) 122. On one hand, YAP enhances mitochondrial OXPHOS efficiency by upregulating mitochondrial leucyl-tRNA synthetase 2, providing metabolic support for TI-Tregs function maintenance 122; on the other hand, YAP amplifies TGF-β/SMAD activation in TI-Tregs by activating key signaling components of the activin receptor complex, inducing forkhead box protein P3 (FOXP3) expression and reinforcing TI-Tregs immunosuppressive function 122, 130. Clinical analyses confirm that infiltration of YAP-high TI-Tregs correlates significantly with poor prognosis in gastric cancer patients 122. In colorectal cancer and melanoma models, elevated matrix stiffness regulates the differentiation and functional maintenance of Tregs through mechanosensitive signaling axes 122. Conversely, reduced YAP activity results in a decreased proportion of FOXP3⁺ Tregs, diminished expression of immunosuppressive molecules, and impaired Treg-mediated immunosuppression, indicating that targeting YAP may overcome Treg-mediated immunotherapy resistance 122, 130.

#### 2.3 Matrix stiffness impairs dendritic cell antigen presentation

DCs play a critical role in antigen presentation and triggering *in vivo* immune responses 131. Immature DCs (iDCs) efficiently recognize and internalize tumor-associated antigens. Upon antigen stimulation, iDCs differentiate into mature DCs (mDCs), characterized by actin cytoskeleton remodeling 132, enhanced migratory capacity, and upregulated expression of MHC molecules and costimulatory molecules such as CD80, CD86 and CD83. These features facilitate antigen presentation and T cells activation, thereby potentiating local anti-tumor immunity 131, 133. However, as a key environmental factor regulating DCs maturation and anti-tumor function, pathologically elevated matrix stiffness not only reduces the number of intratumoral mDCs and impairs their migratory capacity but also disrupts antigen processing and presentation cascades, systematically undermining DCs immunosurveillance. This ultimately attenuates T cell responses and promotes immune evasion 9, 40, 133.

##### 2.3.1 Matrix stiffness suppresses DC antigen uptake and maturation

Matrix stiffness plays a pivotal role in tumor immune evasion by disrupting the initiation of subsequent anti-tumor immune responses through its effects on DC antigen uptake and maturation processes 9, 40, 133. During the antigen recognition and uptake phase, iDCs rely on integrin-mediated dynamic assembly of focal adhesions and podosome extension to monitor the immunogenic microenvironment 134. Elevated stiffness, nevertheless, suppresses tumor immunogenicity by activating the Rho-associated coiled-coil kinase-non-muscle myosin heavy chain ⅡA-filamentous actin pathway in tumor cells, which inhibits cGAS-STING pathway activation and consequently reduces DC differentiation 9. Simultaneously, the physical barrier imposed by stiff matrices limits spatial encounters between iDCs and antigens 40, 135, fundamentally impairing the transition from iDCs to immunocompetent mDCs 9, 40.

Additionally, DCs maturation involves cytoskeletal remodeling and increased cortical stiffness 132, enabling the transition from podosome-rich to amoeboid-like high-velocity migratory phenotypes via podosome disassembly 134, which facilitates lymph node homing. Notably, Morrison *et al.* identified β2 integrin as a negative regulator of DCs maturation, suppressing the mature phenotype by maintaining cytoskeletal linkages and restricting granulocyte-macrophage colony-stimula-ting factor receptor signaling 136. In integrin ligand-coated hydrogel models, elevated matrix stiffness enhances integrin β2-actin mechanical coupling and triggers Lamin A/C-mediated chromatin condensation, which directly suppresses the transcriptional activation of maturation-related genes such as CD86, IL-12, and CC-chemokine receptor 7 (CCR7). Consistent with this, DCs with impaired β2 integrin function exhibit a markedly enhanced maturation profile in melanoma models 133. Consequently, DCs retained within stiff microenvironments undergo maturation arrest, characterized by persistent podosomes that impede the acquisition of an amoeboid migratory phenotype and full immune response activation 134. Jointly, high matrix stiffness-induced dysfunction of DCs facilitates the evasion of immune recognition by tumor cells, thereby promoting tumor immune evasion.

##### 2.3.2 Matrix stiffness impairs DC-mediated T cell activation

Matrix stiffness impairs DC-mediated T cell activation by downregulating surface costimulatory molecules and CCR7 expression on mDCs 133, 137. β2-integrins play a critical role in restricting DC-dependent T cell activation—a perspective inversely validated in studies by Guenther *et al.* 136. Soft substrates (0.87 kPa PA gels), by reducing integrin-mediated mechanical stress, mimic integrin inactivation. Inactive β2 integrins enhance DCs migratory capacity and IL-12 secretion, specifically upregulating expression of costimulatory molecules (CD40, CD80, CD86) and chemokine receptor CCR7 on bone marrow-derived DCs, thereby significantly promoting CD8⁺ T cell infiltration and melanoma suppression 133. Furthermore, in lung adenocarcinoma, the transcription factor SRY-box transcription factor 9 exacerbates tumor matrix stiffening by promoting collagen synthesis, inducing functional impairment of DCs. This manifests as aberrant expression of surface markers including CD103, CD80, CD86, and lysosome-associated membrane protein 3. Such functionally compromised DCs trigger exhausted differentiation of CD8⁺ T cells in the TME, characterized by upregulated lymphocyte activation gene 3 and downregulated Ki67 40, ultimately impairing anti-tumor responses.

Collectively, as a key regulator of DC-mediated immunosuppression, matrix stiffness drives pathological immune evasion through synergistic integrin-dependent mechanosensing defects and transcriptional dysregulation.

#### 2.4 Matrix stiffness suppresses NK cell migration and cytotoxic function

As key effectors of innate immunity, NK cells can detect and destroy cancer cells without the need for tumor-specific recognition or MHC restriction. This mechanism contrasts with that of T cells and endows NK cells with broader antitumor potential 138. This unique biology establishes NK cells as vital components of tumor immunosurveillance 139.

Nevertheless, pathologically elevated matrix stiffness in the TME significantly impairs NK cells cytotoxic activity through synergistic physical constraints and molecular signaling, promoting tumor immune evasion 4, 40. In lung adenocarcinoma, SRY-box transcription factor 9-mediated matrix stiffening correlates significantly with downregulated expression of NK cells functional markers (e.g., granulysin, killer cell lectin-like receptor subfamily D member 1), indicating compromised cytotoxic function 40. This observation is further corroborated in luminal BC: upregulated signal peptide-CUB-EGF domain-containing protein 2 promotes collagen deposition through osteoblast-like differentiation. Collagen fibers—structural determinants of stiffness—are specifically recognized by LAIR1, an inhibitory receptor highly expressed on NK cells. LAIR1-collagen binding triggers SHP-1 phosphorylation cascades that suppress transcriptional expression of effector molecules (IFN-γ, TNF-α) 140.

Furthermore, the activation and effector functions of NK cells require substantial metabolic support from glucose and amino acids such as glutamine and arginine 141. However, in BC, elevated matrix stiffness reprograms the metabolism of TAMs, leading to arginine depletion in the TME 57. This metabolic alteration disrupts glycolytic flux and may induce mitochondrial dysfunction in NK cells, ultimately impairing their cytotoxic activity 141. Through these mechanisms, high tumor matrix stiffness significantly dampens NK cells cytotoxicity and immune reactivity, thereby reducing the efficiency of immune surveillance.

#### 2.5 Matrix stiffness promotes neutrophil N2 polarization and NETs formation

Neutrophils and their NETs are key players in tumor immune regulation 65. Analogous to macrophage plasticity, TANs exhibit environment-dependent phenotypic differentiation: anti-tumor N1-TANs are characterized by high expression of interferon-γ-inducible protein 10/TNF-α and production of reactive oxygen species, whereas pro-tumor N2-TANs upregulate CD206 and CXCL12, secrete higher levels of IL-10, and drive immunosuppression by inhibiting T cell activation and upregulating immune checkpoint molecules 142. Moreover, NETs can form a physical barrier that isolates tumor cells from immune attack and captures circulating tumor cells, thereby promoting immune evasion and diminishing the efficacy of immunotherapy 65, 143. *In vitro* hydrogel models have demonstrated that increased matrix stiffness drives neutrophil N2 polarization and NET formation 144, 145.

Jiang *et al.* demonstrated, using methacrylated gelatin hydrogels that partially recapitulate TME stiffness, that increased matrix stiffness drives neutrophil polarization toward an N2 phenotype via activation of the JAK1/STAT3 mechanotransduction pathway 144. Supporting the relevance of this pathway in tumors, Ozel *et al.* identified STAT3 signaling as a critical regulator of TANs N2 polarization in tumor models, observing that STAT3 knockout significantly suppresses NETs formation while enhancing cytotoxic CD8⁺ T cell-mediated anti-tumor immunity 146. These findings suggest that elevated tumor matrix stiffness may promote immune evasion by regulating N2 polarization and NETosis in neutrophils. Furthermore, Abaricia *et al.* revealed, using polydimethylsiloxane substrates of varying stiffness, that matrix hardness enhances NET formation in a stiffness-dependent manner through integrin/FAK signaling. Although not yet validated in tumor models, this mechanism implies that high stiffness in the tumor stroma may similarly activate neutrophils via the integrin/FAK axis to induce NET formation 145. Collectively, while current evidence—primarily derived from non-tumor models—indicates that matrix stiffness likely influences neutrophil polarization and NET release through multiple mechanisms, further validation in tumor-specific contexts remains essential for future research.

#### 2.6 Matrix stiffness modulates other immune cell populations

Beyond the immune cells previously discussed, the influence of matrix stiffness on other immune populations—such as monocytes and B cells—should not be overlooked. Within the TME, monocytes exhibit dual roles, exerting both anti-tumor effects through the production of anti-tumor effector molecules and activation of antigen-presenting cells 147, as well as pro-tumor functions. Elevated matrix stiffness can skew monocyte function toward a pro-tumor phenotype. For instance, in PDAC, increased matrix stiffness promotes the differentiation of monocytes into M2-like TAMs 11. Furthermore, under 3D high-stiffness conditions, co-culture of estrogen receptor-positive BC cells with monocytes resulted in enhanced monocyte-tumor cell adhesion and increased secretion of pro-tumor proteins, collectively driving the TME toward a tumor-promoting state 148.

B cells enhance anti-tumor immune responses through the production of tumor-specific antibodies, antigen presentation to T cells, and secretion of immunomodulatory molecules. TLS serve as critical hubs within the TME for B cell activation, class switching, and maturation, which are essential for their anti-tumor functions 149. Within TLS, B cells undergo clonal expansion and antibody class switching, leading to the production of high-affinity immunoglobulin G (IgG) and IgA antibodies 150. These antibodies not only directly mediate antibody-dependent cellular cytotoxicity and phagocytosis via Fc receptors on macrophages and NK cells but also form immune complexes that enhance antigen presentation by DCs, thereby amplifying T cell responses and promoting epitope spreading. Additionally, TLS facilitates bidirectional crosstalk between B and T cells, which is critical for sustaining germinal center reactions and plasma cell differentiation 149.

Research indicates that changes in matrix stiffness may have a significant impact on B cell function within the TME. Elevated stiffness has been shown not only to suppress the formation of TLS 24 but also to exert differential regulation on B-cell activation, proliferation, and class-switch recombination 151, 152. Studies by Zeng *et al.* revealed that rigid polydimethylsiloxane substrates enhance the initial phase of B-cell activation by promoting B-cell receptor microcluster formation and recruitment of phosphorylated spleen tyrosine kinase to the immunological synapse, whereas softer substrates preferentially support subsequent clonal expansion, IgM-to-IgG1 class switching, and potent antibody responses *in vivo* 151. Observations in patient-derived breast tumor explant cultures further support the stiffness-dependent promotion of B-cell activation, as soft matrix conditions resulted in a significant reduction in B-cell abundance within the TME 152. We propose that this "double-edged sword" effect reflects an adaptive physiological mechanism, enabling B cells to fine-tune their functional output in response to biomechanical cues to balance rapid infection control against the establishment of antibody diversity and immunological memory 151. During tumor progression, however, persistently elevated stiffness may disrupt this homeostatic balance—not only by impairing TLS assembly 24 but also by skewing B-cell differentiation toward abortive activation, thereby compromising sustained antibody production, inhibiting B-cell recruitment into TLS, and ultimately undermining antitumor immunity.

To summarize, while the role of elevated tumor matrix stiffness in promoting immune evasion through the regulation of immune cells—such as macrophages, T cells, and DCs—has been increasingly elucidated, its specific effects on fundamental cellular behaviors, including activation, proliferation, migration, and secretion, remain highly context-dependent. Owing to substantial variations in substrate materials and culture conditions used across experimental systems, stiffness-induced immunomodulation exhibits considerable heterogeneity, underscoring the complexity of mechanical regulation within the TME. Therefore, there is an urgent need to investigate the spatiotemporal dynamics of matrix stiffness and its regulatory impact on anti-tumor immunity using *in vivo* models and clinical samples.

### 3. Matrix Stiffness Creates a Therapeutic Desert for Immunotherapy

Differing from conventional chemotherapy, radiotherapy, or targeted therapies, immunotherapy operates by "awakening" or "arming" the host immune system to specifically recognize and eliminate tumor cells 153. Despite its substantial therapeutic potential, however, immunotherapy continues to confront significant challenges. Emerging evidence increasingly underscores the critical role of biophysical factors within the TME 4, 154. Among these, elevated matrix stiffness not only establishes physical barriers that hinder the infiltration of immune cells such as T cells, NK cells, and DCs, but also, through modulation of immune cell activation and function, concurrently restricts the transport and penetration of immunotherapeutic agents 7, 155. In aggregate, these effects compromise the efficacy of diverse immunotherapeutic modalities—including ICB, adoptive cell therapy (ACT), and therapeutic cancer vaccines—ultimately impacting clinical treatment outcomes 41, 43**.**

**Figure 5:**
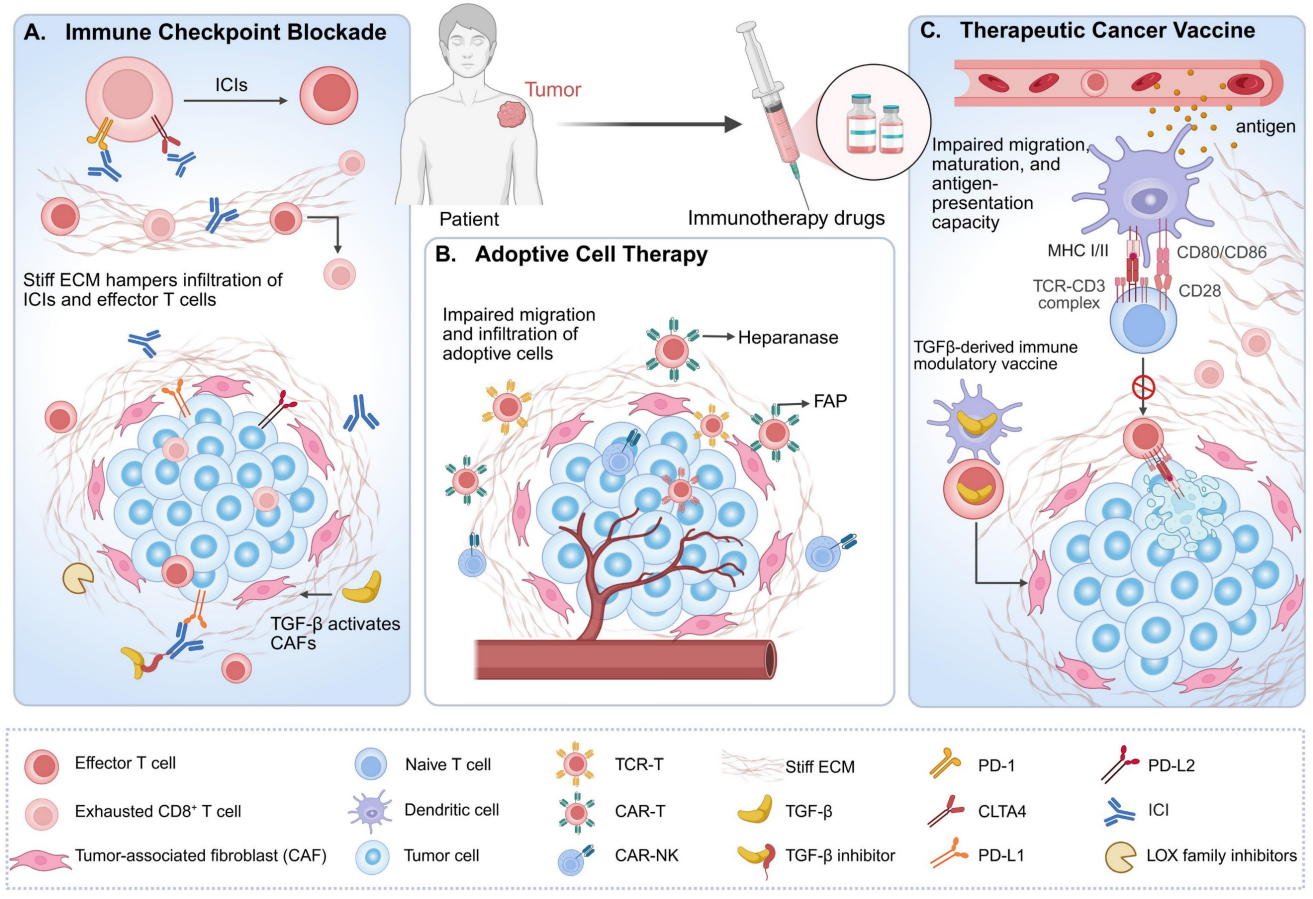
** Elevated matrix stiffness impairs cancer immunotherapy efficacy**. **(A)** Elevated matrix stiffness not only physically impedes the delivery of immune checkpoint inhibitors (ICIs) to exhausted CD8⁺ T cells and deep tumor nests, but may also directly impair the cytotoxic capacity of reinvigorated T cells and promote their re-exhaustion through mechanotransduction signaling. Combining ICIs with inhibitors targeting TGF-β and lysyl oxidase (LOX) family enzymes has emerged as an effective strategy to restore anti-tumor responses. **(B)** This physical barrier similarly hinders the infiltration of adoptive cell therapies (ACTs)—including chimeric antigen receptor T cells (CAR-T), T cell receptor-engineered T cells (TCR-T), and CAR-natural killer (NK) cells—compromising their therapeutic efficacy. Engineering approaches, such as CAR-T cells expressing heparanase or targeting fibroblast activation protein (FAP), have shown promising results in preclinical models by enhancing stromal penetration and specifically targeting stromal cells. **(C)** Elevated matrix stiffness critically impairs the efficacy of therapeutic cancer vaccines by disrupting dendritic cell infiltration, compromising their maturation, and ultimately attenuating their antigen-presenting capacity. Although vaccines can activate and expand antigen-specific T cells capable of recognizing MHC-presented antigens and infiltrating tumors, impaired antigen presentation ultimately attenuates vaccine efficacy. Innovative strategies such as TGF-β vaccines, which enable T cells to directly target cancer-associated fibroblasts (CAFs), offer a novel immunotherapeutic approach.

Significantly, research combining matrix-targeting strategies with immunotherapy is rapidly emerging. Accumulating preclinical evidence consistently indicates that such combinatorial approaches hold promise for enhancing therapeutic efficacy 156-158. However, despite the considerable promise shown by stroma-directed strategies in preclinical models, their clinical translation is complicated by the inherent heterogeneity of ECM components and the critical role of physiological matrix remodeling in normal tissue repair. These factors pose significant challenges to the specificity and safety profiles of such interventions, increasing the risks of off-target effects and unintended toxicity 116. Therefore, there is a pressing need for more rigorously designed studies to evaluate these combination therapies in physiologically and clinically relevant contexts (**Fig. 5**).

#### 3.1 Immune checkpoint blockade therapy

As a cornerstone of cancer immunotherapy, ICB reactivates the anti-tumor immune function of T cells by blocking immune checkpoint molecules such as PD-1/PD-L1, and has emerged as a landmark breakthrough in cancer immunotherapy. Nonetheless, clinical data indicate that only a subset of patients derive benefit from this approach. The mechanisms underlying ICB resistance are multifaceted, with therapeutic responses closely associated with the phenotype of CD8^+^ effector T cells 2. Recent studies have revealed that elevated tumor matrix stiffness not only amplifies cancer immune evasion by upregulating the expression of immune checkpoint molecules PD-L1, PD-1, and cytotoxic T lymphocyte-associated protein 4 (CTLA-4) 43, 159, but also forms physical barriers that impede T cell migration and infiltration 21, 114, while limiting the delivery and penetration of immune checkpoint inhibitors 155. Additionally, it directly exacerbates CD8^+^ T cells exhaustion and downregulates their cytotoxicity via mechanotransduction pathways 37, synergistically contributing to ICB treatment failure. For instance, partial non-response to anti-CTLA-4 therapy stems from collagen deposition-induced reductions in T cells tumor infiltration 160. Recent investigations have further demonstrated that matrix stiffness can induce PD-L2 expression and impair the efficacy of sorafenib combined with anti-PD-1 therapy in treating HCC *in vivo* 161.

In response, a growing body of preclinical evidence supports the combination of matrix stiffness-targeting agents with ICB 156, 162. For example, a nanoscale remodeler (SPNcb) that integrates photodynamic therapy with tumor-specific LOX inhibition enables activatable cancer photoimmunotherapy. By reducing matrix stiffness, it enhances drug penetration and immune cell infiltration, and when combined with anti-PD-L1 checkpoint blockade, significantly improves antitumor efficacy 155. Similarly, TGF-β targeting suppresses TGF-β signaling in CAFs and reduces ECM deposition, thereby alleviating both physical and immunosuppressive barriers to T-cell infiltration in the TME. This approach synergizes with PD-L1 inhibition to enhance antitumor immune responses in BC models 162.

These findings suggest that targeted modulation of matrix stiffness to improve T cells function may emerge as a novel strategy to overcome ICB resistance, underscoring the critical clinical translational value of elucidating the interplay between matrix mechanical properties and immunotherapy.

#### 3.2 Adoptive cell therapy

ACT represents a primary modality of cancer cell-based treatment. Its core mechanism involves extracting immune cells from patients, enhancing their anti-cancer capabilities through genetic modification or ex vivo expansion, and re-infusing them into the patient. Major ACT subtypes include CAR-T therapy, T cell receptor-engineered T-cell therapy, tumor-infiltrating lymphocytes therapy, and NK cell therapy 163. Nonetheless, the efficacy of ACT is highly dependent on the infiltration capacity, persistence, and functional maintenance of immune effector cells within the TME—key processes that are significantly regulated by matrix stiffness 41, 157.

Studies have demonstrated that increased matrix stiffness not only impedes tumor penetration by T cells and NK cells while dampening their anti-tumor responses 39, 40, but also severely restricts the migration and infiltration efficiency of adoptively transferred cells within the TME, emerging as a primary bottleneck in ACT for solid tumor treatment 157, 164. For instance, physical barriers formed by the tumor ECM hinder the migration of CAR-T cells to target sites, thereby reducing their clinical efficacy 157, 164.

To address this challenge, modulating TME mechanical properties to enhance adoptive cell infiltration and anti-tumor immune function has emerged as a breakthrough strategy 164, 165. Recent studies, using a human BC MDA-MB-231 xenograft model, have demonstrated that intratumoral pretreatment with nattokinase effectively reduces matrix stiffness, significantly enhances CAR-T cells tumor infiltration, and potentiates their tumor-suppressive effects—offering novel insights for improving the clinical efficacy of ACT 165. In addition to enzymatic approaches, alternative strategies—such as engineering CAR-T cells to express heparanase—enhance their capacity to degrade the ECM, thereby facilitating T-cell infiltration and augmenting anti-tumor activity 164. Furthermore, combining thermally inducible CAR-T cells with hyperthermia regimens mitigates the desmoplastic characteristics of stiff TMEs and improves T-cell penetration through the ECM, ultimately enhancing the efficacy of CAR-T cell therapy in glioblastoma 157.

These ongoing explorations provide valuable insights into targeting the tumor ECM to optimize ACT, highlighting a dynamic and rapidly evolving research landscape with significant implications for developing more effective cancer immunotherapies.

#### 3.3 Therapeutic cancer vaccines

Fundamental principles governing successful therapeutic cancer vaccination encompass the delivery of high-quality antigens to DCs in substantial quantities, optimal DCs activation, induction of robust and sustained CD4^+^ T helper cell and cytotoxic T lymphocyte (CTL) responses, as well as the persistence of TME infiltration and reactivity 166. Nevertheless, elevated matrix stiffness disrupts this immunological cascade through multifaceted mechanisms: it not only impedes DCs tumor infiltration and maturation processes but also significantly impairs their antigen presentation capacity, ultimately resulting in inadequate T cells activation and defective anti-tumor immune responses 137, 167. These effects severely limit the efficacy of therapeutic cancer vaccines.

To overcome these barriers, innovative strategies continue to emerge. Hu *et al.* combined a tumor nanovaccine with a sperm adhesion molecule 1-mediated ECM-clearing agent, which promoted immune cell infiltration and achieved potent antitumor efficacy in B16-OVA tumor-bearing mice 158. Similarly, Perez-Penco *et al.* developed a TGF-β vaccine that reduces immunosuppressive M2-like TAMs and myofibroblastic CAFs, thereby alleviating immune exclusion and matrix stiffness in the pancreatic cancer TME. This approach has shown promise in preclinical models and is currently under evaluation in a Phase Ⅰ clinical trial (EudraCT No. 2022-002734-13) 168. These advances underscore the promising potential of targeting stromal biomechanics to enhance the efficacy of tumor vaccines and restore antitumor immunity.

### 4. Matrix Stiffness-Targeted Therapeutic Strategies

As the tumor mechanical microenvironment emerges as a novel cancer hallmark, targeting this biomechanical niche has become an innovative therapeutic paradigm. Current cancer immunotherapy strategies primarily focus on enhancing anti-tumor immune responses by modulating biochemical signaling between tumor and immune cells. The emerging concept of "mechano-immune surveillance," however, has opened new avenues for developing next-generation therapeutic strategies targeting the biophysical properties of immune cells 5. Reducing matrix stiffness, for instance, enhances T cell migration and potentiates the efficacy of anti-PD-1 therapy 116. Nanoparticle-based systems, such as H/S@hNP, utilize a cholesterol depletion strategy to soften the tumor stroma, enhance deep drug penetration, and reprogram TAMs from an M2 to an M1 phenotype, thereby augmenting photodynamic therapy and overall antitumor responses 169. Importantly, targeting the tumor ECM—by reducing collagen density, increasing fiber porosity, and promoting structural loosening—facilitates the formation and maturation of TLS 24. These organized lymphoid aggregates promote antitumor immunity within the TME by coordinating the activation of T and B cells, including the enhancement of CTL responses and the production of tumor-specific antibodies. Their presence is associated with improved clinical outcomes and favorable responses to immunotherapy 29.

Therefore, targeting matrix stiffness to improve drug delivery, immune cell infiltration, and activation holds critical clinical significance in augmenting immunotherapeutic outcomes 32. Current intervention approaches primarily involve matrix-modifying agents that degrade ECM components or inhibit their biosynthesis to reduce matrix stiffness 18. However, studies indicate that combination strategies integrating such matrix-modulating agents with immunotherapy can yield superior therapeutic outcomes. For instance, engineered CAR-T cells endowed with ECM-degrading capacity exhibit enhanced antitumor efficacy 164 and may facilitate the adhesion and infiltration of other immune cells within stiffened matrices. Based on the dynamic interplay between matrix stiffness and immune cell behavior, we propose targeting key drivers of stromal stiffening—such as CAFs, TGF-β, LOXs, and MMPs—to disrupt the biomechanically aberrant TME and reverse immunosuppression. Combining these agents with existing immunotherapies can synergistically disrupt matrix biomechanical properties and overcome immune evasion mechanisms, heralding new breakthroughs in cancer treatment.

#### 4.1 Reduction of matrix deposition

##### 4.1.1 Targeting the principal architect CAFs

As the primary architects of the desmoplastic reaction and subsequent high matrix stiffness in tumors, CAFs have emerged as a highly attractive therapeutic target 26, 170. Targeting CAFs via nanomedicine strategies offers a promising avenue for treating desmoplastic malignancies, enabling both direct tumor cell killing and remodeling of the TME 171. Although CAF-targeted therapies hold great potential for degrading the ECM, dismantling physical barriers, and enhancing immunotherapeutic efficacy, their development has been hampered by a lack of specific biomarkers and marked functional heterogeneity. Among potential targets, fibroblast activation protein (FAP) has surfaced as one of the most promising candidates 170.

Preclinical studies have demonstrated that multiple FAP-targeted therapeutic modalities—including DNA vaccines and CAR-T cells—effectively eliminate FAP⁺ CAFs, disrupt stromal integrity, reduce collagen deposition, and enhance CD8⁺ T-cell infiltration, thereby overcoming immune exclusion and augmenting antitumor immunity and therapeutic efficacy 25, 172. Notably, this FAP-dependent stromal remodeling synergizes with anti-PD-1 therapy, enhancing CD8⁺ T-cell function and reducing PD-1 expression 25. For example, the FAP-targeting antibody-drug conjugate OMXT705 specifically engages FAP⁺ CAFs and releases the cytotoxin TAM470 to kill tumor cells. Even in PD-1-resistant models, OMXT705 promotes immune cell infiltration 173. These compelling preclinical findings are corroborated by human data: a first-in-human study of the FAP-directed 4-1BB agonist RO7122290 (alone or combined with atezolizumab) showed that combination therapy more than doubled intratumoral CD8⁺ T-cell infiltration in over half of the patients compared to monotherapy (**Table 1**). Furthermore, an objective response rate of 10% was observed in immune checkpoint inhibitor-naïve patients, confirming immune activation via stromal modulation 174.

Together, these cumulative findings solidify FAP⁺ CAFs as a key target for disrupting matrix stiffness and enhancing antitumor immunity. Combining FAP inhibition with immunotherapy represents a rational strategy to improve response rates and provides a clear direction for future clinical translation.

##### 4.1.2 Targeting TGF-β-mediated matrix deposition

TGF-β significantly promotes the synthesis and deposition of ECM by activating CAFs, positioning it as a potential therapeutic target for inhibiting stromal matrix production 61. By specifically targeting TGF-β, it is possible not only to remodel the ECM but also to optimize the TME, thereby facilitating more effective therapeutic outcomes 61, 175. For instance, normalization of the ECM via TGF-β inhibitors has been shown to significantly enhance the delivery efficiency of chemotherapeutic and nanomedicines within tumors, thereby improving their anti-tumor efficacy 176. Concurrent treatment with TGF-β-blocking antibodies and anti-PD-L1 antibodies reduces TGF-β signaling in stromal cells, promotes T cell infiltration into the tumor core, and elicits robust anti-tumor immunity and tumor regression 162.

To date, numerous TGF-β-targeted agents have been developed, including small-molecule inhibitors, monoclonal antibodies, and vaccines 168, 177. Pirfenidone, a clinically approved anti-fibrotic drug targeting TGF-β, has been shown to significantly reduce collagen area fraction and matrix stiffness in orthotopic breast tumor models 178. Additionally, the bifunctional fusion protein bintrafusp alfa (M7824), which simultaneously targets TGF-β and PD-L1, demonstrated promising results in Phase I trials for advanced solid tumors 179 and Phase II trials for cervical cancer 180; however, Phase II trials in head and neck squamous cell carcinoma (NCT04428047) and triple-negative BC (NCT04489940) were terminated due to safety concerns or suboptimal efficacy. In contrast, multiple innovative therapeutic strategies are currently being explored to target TGF-β in combination approaches. For instance, the clinical trial of ficerafusp alfa (BCA101) in combination with pembrolizumab for PD-L1-positive head and neck squamous cell carcinoma (NCT06788990) is currently ongoing. Additionally, the development of TGF-β-based vaccines offers a novel strategy to address immune exclusion and matrix stiffening in the PDAC TME. By modulating the phenotypes of myofibroblastic CAFs and M2-like TAMs, this approach reduces the physical barrier posed by stiffened stroma and alleviates the immunosuppressive microenvironment, thereby improving immune cell migration and infiltration within rigid matrices. A Phase I clinical trial (EudraCT No. 2022-002734-13) evaluating this strategy is currently underway 168.

Overall, these advances highlight continued efforts to optimize combinatorial therapeutic regimens targeting TGF-β signaling. Exploration of other TGF-β-targeted immunotherapeutic agents remains in its early stages, necessitating extensive clinical trials to evaluate the safety and efficacy of these potential inhibitors 43 (**Table 1**).

#### 4.2 Targeting LOX-mediated matrix crosslinking

Matrix crosslinking mediated by LOXs serves as a critical contributor to elevated tumor matrix stiffness, positioning LOXs as key therapeutic targets for matrix regulation 181. LOX inhibitor-mediated reduction of matrix stiffness has been shown to significantly enhance T cell tumor infiltration and migratory capacity, while synergistically potentiating the anti-tumor efficacy of PD-1/PD-L1 blockade therapy. These findings provide crucial evidence supporting the clinical translation of LOX inhibitor combinations with immune checkpoint inhibitors 43, 116.

β-aminopropionitrile, the first widely used non-specific LOX family inhibitor, exerts its effects by irreversibly binding to the enzyme's active site. Studies in multiple tumor models (e.g., MMTV-PyMT BC, KPC pancreatic cancer, and EGI-1 cholangiocarcinoma) have confirmed its ability to effectively mitigate collagen crosslinking-induced matrix stiffening and notably improve T cell migration 116. Nevertheless, its use in clinical trials was halted due to high toxicity. In contrast, the monoclonal antibody AB0023 targeting LOXL2 demonstrates superior safety and efficacy: it simultaneously suppresses tumor metastasis and TGF-β signaling activation, showing broad applicability in both oncologic and fibrotic diseases 182. Additionally, novel oral aminomethy-lthiophene-based compounds, acting as dual LOX/LOXL2 inhibitors, have emerged as promising candidates due to their excellent bioavailability and anti-tumor activity 183.

While LOX inhibitors show promising potential in fibrotic disease trials (NCT04676529, NCT04054245) 184 (**Table 1**), their application in malignant tumor treatment remains in the exploratory stage, necessitating urgent advancement of related clinical investigations.

#### 4.3 Targeting MMP-mediated pathological ECM degradation

MMPs represent the primary enzyme family responsible for degrading collagen and other proteins within the ECM, thereby facilitating tumor cell dissemination 76. Although the therapeutic rationale for MMP inhibition is well-founded, early broad-spectrum MMP inhibitors—such as batimastat and marimastat—suffered from insufficient specificity and severe off-target effects due to disruption of physiological ECM remodeling. These limitations ultimately resulted in significant dose-limiting toxicities, greatly impeding their clinical translation 185.

The advent of nanotechnology has brought a paradigm shift to MMP-targeting strategies. By enabling targeted delivery and significantly reducing systemic toxicity, novel nanoformulations have effectively overcome the historical limitations of conventional inhibitors. This advancement has not only revitalized the therapeutic potential of MMP inhibition but also opened new avenues for utilizing MMPs as both tumor biomarkers and druggable targets 186. For instance, an MMP-2/pH dual-sensitive, hierarchically structured nanoparticle platform was engineered to be size-switchable, dynamically balancing tumor accumulation and deep tissue penetration. This multifunctional strategy enhances nanodrug penetration across ECM barriers and significantly improves antitumor efficacy 187. Meanwhile, the MMP-9 inhibitor HY-135232 effectively reverses Epstein-Barr virus-induced M2 macrophage-mediated suppression of T-cell receptor-engineered T cells. The combination of these agents offers a promising synergistic approach for treating EBV-positive solid tumors 188.

Currently, a clinical trial (NCT04214392) (**Table 1**) is evaluating the safety and feasibility of chlorotoxin-directed CAR-T cells targeting MMP-2 in patients with glioblastoma 189. Nevertheless, the clinical development of MMP inhibitors remains underexplored. High structural homology and functional redundancy among MMP subtypes continue to pose challenges for achieving selective inhibition, underscoring the need for future efforts to develop subtype-specific inhibitors or context-dependent targeting strategies 81.

### 5. Conclusions and Perspectives

Immune evasion, a hallmark of cancer, emerges during the initial stages of tumor development and serves as a critical driver of sustained tumor growth 3. In the early phase, immune surveillance predominates, characterized by robust infiltration of anti-tumor immune cells and a modest increase in matrix stiffness that remains near physiological levels. Interactions between immune cells and the ECM primarily involve immune recognition of ECM antigens and participation in the clearance of transformed cells. Concurrently, nascent tumor cells initiate early immune evasion mechanisms, including upregulation of immune checkpoint molecules such as PD-L1, limited recruitment of immunosuppressive cells like Tregs, and alterations in the local metabolic microenvironment. In contrast, the late stage is marked by a profound shift in the co-evolutionary dynamics within the TME, where immuno-suppression becomes dominant. This transition is evidenced by a reduction in cytotoxic CD8⁺ T cells and NK cells, impaired maturation and function of DCs, and expansion of immunosuppressive cell populations 3, 190. Importantly, the persistent activation of CAFs and dysfunctional immune cells, among other factors, collectively drive extensive ECM remodeling, resulting in a marked increase in matrix stiffness 20, 26, 32, 51, 62. This process not only creates a physical barrier that impedes immune cell infiltration but also actively suppresses immune cell function through mechanotransductive signaling, ultimately fostering an immunosuppressive TME conducive to immune evasion 38, 39, 100.

This pathological elevation of matrix stiffness is now recognized as one of the most prominent biomechanical features of the TME, playing a critical role in driving both tumor progression and immune evasion. Rather than acting unidirectionally, matrix stiffness and immune cells engage in a vicious cycle that effectively promotes tumor immune evasion. In this review, we propose the "matrix stiffness-immune cell bidirectional regulatory axis" to conceptualize this loop: (1) Immune cells—including TAMs, TANs, and B cells—promote increased secretion, excessive deposition, and aberrant cross-linking of the ECM through direct secretion of ECM components and cross-linking enzymes (e.g., LOXs), or by activating CAFs via paracrine signals such as TGF-β. These processes collectively contribute to elevated matrix stiffness; (2) Elevated matrix stiffness in turn impairs the cytotoxicity of effector cells (e.g., CD8⁺ T cells and NK cells) via mechanosensors (e.g., integrins, mechanosensitive channels) and downstream effectors (e.g., YAP/TAZ, JAK/STAT), disrupts antigen presentation by DCs, and promotes the polarization, recruitment, and immunosuppressive activity of inhibitory immune cells; (3) This reciprocal crosstalk establishes a self-sustaining immuno-suppressive microenvironment in which dysfunctional immune cells exacerbate matrix stiffness, which in turn further aggravates immune cell dysfunction through persistent mechanotrans-ductive signaling. This profound interaction represents a key mechanism underlying immune evasion and therapy resistance.

In addition to the mechanotransduction-mediated regulation of immune cell behavior by matrix stiffness, biochemical signals also play synergistic roles 36, 57. A representative example is the mutually reinforcing interaction between TGF-β and matrix stiffness. Specifically, TGF-β activates CAFs via the canonical SMAD signaling pathway, promoting excessive ECM deposition and consequently increasing matrix stiffness 61, 63. This mechanical enhancement, in turn, augments the sensitivity of TAMs to TGF-β through integrin-mediated mechanosensing, facilitating M2 polarization and further promoting the secretion and activation of TGF-β1 48, 57. This process establishes a positive feedback loop termed the "stiffness-TGF-β-TAM" axis. Moreover, matrix stiffness can cooperate with other biochemical signals, such as IL-4 and IL-13, by enhancing chromatin accessibility to amplify the transcriptional efficiency of the JAK-STAT6 pathway downstream of the IL-4 receptor, thereby collectively driving M2 macrophage polarization 36, 90. Although these interactions are only briefly outlined here, their functional significance extends beyond the current discussion, underscoring the urgent need for future research to dissect the synergistic mechanisms by which biochemical cues and mechanical signals collectively shape immune cell fate and function.

Targeting matrix stiffness is emerging as a critical combinatorial strategy to overcome the limitations of current immunotherapy. By elucidating the dynamic interplay between matrix stiffness and immunity, we propose that combining therapies targeting key drivers of ECM remodeling—such as CAFs, TGF-β, LOXs, and MMPs—with immunotherapeutic agents provides a compelling scientific rationale for the clinical translation of mechano-immunotherapeutic strategies. A key frontier in translating these therapeutic approaches into clinical practice is the development of reliable predictive and pharmacodynamic biomarkers. Promising candidate biomarkers include circulating enzymes directly involved in matrix remodeling, such as LOXL2, whose levels correlate with stromal content, metastasis, and poor survival 191. Other potential circulating biomarkers comprise additional cross-linking enzymes (e.g., PLOD2^192^) and MMPs 193. Beyond liquid biopsies, imaging biomarkers hold substantial potential for providing direct spatial assessment of intratumoral mechanics in patients. Techniques such as magnetic resonance elastography and ultrasound shear-wave elastography enable non-invasive mapping of tissue stiffness, offering tools for patient stratification and treatment response monitoring 194, 195. Future efforts should focus on validating these biomarkers in prospective clinical trials and integrating multi modal data (circulating + imaging) to construct comprehensive predictive models.

Despite the promising diagnostic (as biomarkers) and therapeutic potential of targeting matrix stiffness, its clinical translation faces significant challenges due to the complex role of the ECM in tumor progression and its dynamic remodeling nature. In response, emerging mechano-immuno-therapeutic approaches are attracting increasing attention. Nanotechnology, for instance, offers an innovative platform for ECM-based cancer therapy by enabling spatiotemporally programmable drug delivery with enhanced precision and sustainability. Functionalized nanoparticles allow controlled release at specific targets and cellular subtypes, and can be activated by external energy sources to initiate multimodal antitumor responses 196, 197. Looking forward, the use of alternating magnetic field-driven magnetic nanoparticles may achieve localized hyperthermia within the TME to modulate regional matrix stiffness, while real-time imaging provides feedback on thermal dose and distribution—paving a novel path toward optimized personalized treatment. Furthermore, 3D bioprinting techniques utilizing bioinspired ECM hydrogels have advanced the development of physiologically relevant tumor models. For example, gelatin methacryloyl-based models of gastric cancer can recapitulate clinical disease features 198. These advanced systems provide powerful platforms for elucidating immune cell-ECM interactions and enabling high-throughput screening of novel mechanosensitive therapeutic agents.

The continued evolution of these mechano-immunotherapeutic strategies offers transformative opportunities to address long-standing challenges at the intersection of biomechanics and tumor immunology. Although still in the exploratory stage, mechano-immunotherapy introduces a paradigm shift in cancer treatment, propelling the field toward more precise and effective interventional strategies. Future research should pivot toward innovative, rigorously designed clinical trials evaluating the therapeutic efficacy of stiffness-targeting interventions combined with immunotherapies, promising to offer novel solutions for overcoming clinical immunotherapy resistance. Ultimately, deepening our understanding of the "matrix stiffness-immune cell bidirectional regulatory axis" will not only revolutionize knowledge of tumor immune evasion mechanisms but also catalyze the development of next-generation combinatorial immunotherapies targeting TME biomechanics, bringing new hope for breakthroughs in precision oncology.

## Figures and Tables

**Table 1:** Clinical trials of drugs targeting matrix stiffness (2020-2025) (clinicaltrials.gov).

Target	Category	Drug	Combination partner	Diseases	Phase	Primary endpoints	NCT number	Status
TGF-β	Anti-PD-L1 and TGF-β bifunctional fusion protein	Bintrafusp Alfa (M7824)	Monotherapy	Cervical Cancer	II	ORR	NCT04246489 Ref. 180	Completed
			+Paclitaxel, Carboplatin, External beam radiotherapy	Squamous Cell Carcinoma of the Esophagus or Gastroesophageal Junction	None	Feasibility study	NCT04481256	Recruiting
			Monotherapy	Malignant Pleural Mesothelioma	II	PFS	NCT05005429	Completed
	TIGIT/TGF-β bifunctional fusion protein	AK130	Monotherapy	Malignant Tumors	I	Safety and tolerability, severity of adverse events	NCT05653284	Completed
	Anti-PD-L1 and TGF-β bifunctional fusion protein	HB0028	Monotherapy	Solid Tumors	I/II	Safety and tolerability	NCT06223308	Recruiting
	Anti-PD-L1 and TGF-β bispecific antibody	Y101D	Monotherapy	Solid Tumors	I	Safety and tolerability	NCT05028556	Completed
			+Gemcitabine, Albumin paclitaxel	Pancreatic Cancer	Ib/II	Safety and efficacy, ORR	NCT06266143	Active, not recruiting
	Targeting CD39 and TGF-β dual antidrugs	ES014	Monotherapy	Solid Tumors	I	Safety and tolerability, severity of adverse events	NCT05717348	Active, not recruiting
				Solid Tumors	II	ORR	NCT06543056	Recruiting
	Anti-PD-L1 and TGF-β fusion protein	HCB301	Monotherapy	Advanced Tumors	I	Safety and tolerability	NCT06487624	Recruiting
	Dual function EGFR/TGF-β antibody	Ficerafusp Alfa (BCA101)	+Pembrolizumab	Head and Neck Squamous Cell Carcinoma (HNSCC)	II/Ⅲ	Safety and tolerability, OS, ORR	NCT06788990	Recruiting
	Bifunctional TGF-β antagonist/IL-15 protein complex	HCW9218	Monotherapy	Pancreatic Cancer	Ib/II	Safety and tolerability, severity of adverse events	NCT05304936	Active, not recruiting
				Solid Tumors	I	Safety and tolerability	NCT05322408	Active, not recruiting
	Anti-PD-L1 and TGF-β recombinant bifunctional molecule	BJ-005	Monotherapy	Solid Tumor or Lymphoma	I	Safety and tolerability, severity of adverse events	NCT05115292	Active, not recruiting
	An anti-TIGIT IgG1 monoclonal antibody	PM1021	Monotherapy or + PM8001	Solid Tumours	I	Safety and tolerability, severity of adverse events	NCT05537051	Not yet recruiting
	A dual-target CAR-T cell therapy with TGF-β resistance and a safety switch	RD133	Monotherapy	MSLN-Positive Solid Tumors	II	Safety and tolerability, severity of adverse events	NCT05141253	Recruiting
TGF-β1	TGF-β1 blocker	losartan (LOS)	Monotherapy	RIF in the Breast and the Lung of Breast Cancer Patients	II	Fibrosis severity grade	NCT05637216	Recruiting
			+FOLFIRINOX, 9-ING-41	Pancreatic Cancer	II	PFS	NCT05077800	Active, not recruiting
TGF-β2	TGF-β2 inhibitor	OT-101	+Pembrolizumab	Malignant Pleural Mesothelioma	II	ORR	NCT05425576	Not yet recruiting
TGF-βRI	TGF-β1 receptor inhibitor	Vactosertib (TEW-7197)	Monotherapy	Osteosarcoma	I/II	Safety and tolerability, severity of adverse events	NCT05588648	Recruiting
TGF-βRII	An anti-PD 1/TGF-βRII bifunctional fusion protein	TQB2868	+Gemcitabine injection, Albumin paclitaxel injection, Anlotinib capsules	Metastatic Pancreatic Neoplasms	II	PFS	NCT06767813	Recruiting
LOXs	Pan-LOX/LOXL inhibitor	PXS-5505	Monotherapy	Myelofibrosis	I/IIa	Safety and tolerability	NCT04676529	Completed
FAP	A FAP targeted CD40 agonist	RO7300490	Monotherapy or + Atezolizumab	Solid Tumors	I	Safety and tolerability, ORR	NCT04857138	Completed
	A bispecific fusion protein targeting FAP and 4-1BB	RO7122290	Monotherapy or + Atezolizumab	Solid Tumors	I	Safety and tolerability, DC, ORR	None Ref. 174	Completed
		RO7122290	+Obinutuzumab	Metastatic Colorectal Cancer	Ib	Safety and tolerability	NCT04826003	Completed
	An anti-FAP ADC	OMTX705	Monotherapy or + Pembrolizumab	Advanced Solid Tumor	I	Safety and tolerability	NCT05547321 Ref. 173	Recruiting
	A FAP targeted CD40 agonist	RO7300490	Monotherapy or + Atezolizumab	Solid Tumors	I	Safety and tolerability, ORR	NCT04857138	Completed
	A FAP dependent CD40 agonist	MP0317	+Gemcitabine, Cisplatine, Durvalumab	Advanced Biliary Tract Carcinoma	II	PFS	NCT07036380	Not yet recruiting
MMP-2	Chlorotoxin-conjugated CAR T cells	Chlorotoxin-directed CAR T cells	Monotherapy	Glioblastoma	I	Safety and tolerability	NCT04214392 Ref. 189	Active, not recruiting

ADC: antibody-drug conjugate; CAR-T: chimeric antigen receptor T cell therapy; EGFR: epidermal growth factor receptor; FAP: fibroblast activation protein; IgG: immunoglobulin G; IL-15: interleukin-15; LOX: lysyl oxidase; LOXL: lysyl oxidase-like; MMP: matrix metalloproteinase; ORR: objective response rate; OS: overall survival; PFS: progression-free survival; PD-L1: programmed death-ligand 1; TGF-β: transforming growth factor-β; TGFβR: transforming growth factor-β receptor; TIGIT: T-cell immunoreceptor with Ig and ITIM domains.

## Abbreviations

3D: three-dimensional
ACT: adoptive cell therapy
ADC: antibody-drug conjugate
AKT: protein kinase B
AMPK: AMP-activated protein kinase
ATP: adenosine triphosphate
BC: breast cancer
CAF: cancer-associated fibroblasts
CaMKII: Ca²⁺/calmodulin-dependent protein kinase II
CaMKK2: Calcium/calmodulin-dependent protein kinase kinase 2
CAR-T: chimeric antigen receptor T cell
CCL7: C-C motif chemokine ligand 7
CCR7: C-C chemokine receptor 7
CREB: cAMP response element-binding protein
CTL: cytotoxic T lymphocyte
CTLA-4: cytotoxic T lymphocyte-associated antigen 4
CXCL3: C-X-C motif chemokine ligand 3
CXCR2: C-X-C chemokine receptor 2
DC: dendritic cell
ECM: extracellular matrix
ERK1/2: extracellular signal-regulated kinase 1/2
FAK: focal adhesion kinase
FAP: fibroblast activation protein
FGF: fibroblast growth factor
FOXP3: forkhead box protein P3
GM-CSF: granulocyte-macrophage colony-stimulating factor
HCC: hepatocellular carcinoma
HDAC3: histone deacetylase 3
HIF-1α: hypoxia-inducible factor 1-α
HSPG2: heparan sulfate proteoglycan 2 (Perlecan)
ICAM-1: intercellular adhesion molecule 1
ICB: immune checkpoint blockade
ICIs: immune checkpoint inhibitors
iDC: immature dendritic cell
IFN-γ: interferon-γ
IgG: immunoglobulin G
IL: interleukin
IQGAP1: IQ motif-containing GTPase-activating Protein 1
PYK2: proline-rich tyrosine kinase 2
IS: immune synapse
JAK: Janus kinase
KLRD1: killer cell lectin-like receptor subfamily D member 1
LAG-3: lymphocyte-activation gene 3
LAIR1: leukocyte-associated immunoglobulin-like receptor-1
LAMP3: lysosomal-associated membrane protein 3
Lars2: Lars2, leucyl-tRNA synthetase 2, mitochondrial
LFA-1: lymphocyte function-associated antigen 1
LINC: linker of nucleoskeleton and cytoskeleton
LOX: lysyl oxidase
LOXL: lysyl oxidase-like
mDC: mature dendritic cell
MEK1/2: mitogen-activated protein kinase kinase 1 and 2
MHC: major histocompatibility complex
MMP: matrix metalloproteinase
NETs: neutrophil extracellular traps
NFAT1: nuclear factor of activated T-cells 1
NF-κB: nuclear factor kappa-light-chain-enhancer of activated B cells
NK cell: natural killer cell
ORR: objective response rate
OS: overall survival
Osr2: odd-skipped related transcription factor 2
OXPHOS: oxidative phosphorylation
P4HA1: prolyl 4-hydroxylase subunit α1
PA: polyacrylamide
PCOLCE: procollagen C-endopeptidase enhancer
PD-1: programmed cell death protein 1
PDAC: pancreatic ductal adenocarcinoma
PD-L1: programmed death-ligand 1
PDGF: platelet-derived growth factor
PFS: progression-free survival
Piezo1: Piezo type mechanosensitive ion channel component 1
PI3K: phosphatidylinositol 3-kinase
PLOD: procollagen-lysine, 2-oxoglutarate dioxygenase
Prf1: perforin-1
RELA: v-rel reticuloendotheliosis viral oncogene homolog A
SHP-1: Src homology region 2 domain-containing phosphatase-1
STAT: signal transducer and activator of transcription
TAM: tumor-associated macrophage
TAN: tumor-associated neutrophil
TCR: T cell receptor
TEAD: TEA domain transcription factor
TGF-β: transforming growth factor-β
TIM-3: T cell immunoglobulin and mucin domain-containing protein 3
TIMP-1: tissue inhibitor of metalloproteinases-1
TI-Tregs: tumor-infiltrating regulatory T cells
TLS: tertiary lymphoid structure
TME: tumor microenvironment
TNF-α: tumor necrosis factor-α
Treg: regulatory T cell
TRPV4: transient receptor potential vanilloid 4
YAP: Yes-associated protein
